# PTH counteracts Hippo signaling via Src-dependent YAP stabilization to enhance bone marrow stromal cell differentiation

**DOI:** 10.1172/jci.insight.191245

**Published:** 2025-07-22

**Authors:** Sara Monaci, Mengrui Wu, Hiroyuki Okada, Kedkanya Mesil, Byeong-Rak Keum, Maisa Monseff Rodrigues da Silva, Clifford J. Rosen, Francesca Gori, Roland Baron

**Affiliations:** 1 Department of Oral Medicine, Infection, and Immunity, Harvard School of Dental Medicine, Boston, Massachusetts, USA.; 2 MaineHealth Institute for Research, Maine Medical Center, Scarborough, Maine, USA.; 3 Department of Medicine, Harvard Medical School, Boston, Massachusetts, USA.

**Keywords:** Bone biology, Endocrinology, Osteoclast/osteoblast biology

## Abstract

Parathyroid hormone (PTH) regulates serum calcium and phosphate through its actions in bone and kidney and is used to increase bone in osteoporosis treatment. In bone, PTH targets osteoblasts and osteocytes to regulate bone remodeling but also bone marrow stromal cells (BMSCs), regulating their differentiation in the osteoblast or the adipocyte lineage. PTH exerts its action through the PTH/PTH-related peptide (PTHrP) receptor, a G protein–coupled receptor (GPCR), activating adenylyl cyclase and phospholipase C (PLC). Although the effects of cAMP and PKA are well characterized, little is known about the effects of PLC activation or on the crosstalk between PTH signaling and other pathways. Here, bulk RNA-Seq of PTH-treated murine BMSC line (W-20) revealed significant changes in the Hippo pathway. In addition to increasing its transcription, PTH stabilized YAP protein, a key target of Hippo, by decreasing YAP/LArge Tumor Suppressor kinase 1 (LATS1) interaction, YAP^S127^ phosphorylation, and YAP ubiquitination, leading to YAP nuclear translocation and expression of YAP target genes. Similar events occurred in osteocyte cell lines. This occurred via an increase in Src kinase activity: We identified YAP^Y428^ as a key tyrosine residue phosphorylated by Src in response to PTH. Preventing YAP^Y428^ phosphorylation led to YAP instability, blocking both osteogenic and adipogenic differentiation of W-20 cells. These results demonstrate active crosstalk between the PTH/PTHrP and the Hippo signaling pathways and reveal that PTH signaling utilizes the PLC/Ca^2+^/Src tyrosine kinase signaling cascade to influence YAP stability, antagonizing Hippo signaling and favoring stromal cell differentiation. Thus, PTH signaling counteracts the effects of Hippo signaling in BMSCs to favor their differentiation.

## Abstract 2

PTH signaling negatively regulates the Hippo pathway in bone marrow stromal cells via YAP stabilization by Src tyrosine phosphorylation, favoring BMSC differentiation.

## Introduction

Parathyroid hormone (PTH), an 84–amino acid peptide secreted from the parathyroid glands, regulates serum calcium and phosphate levels through its direct actions in bone and kidney. In bone, PTH targets osteoblasts and osteocytes to increase directly bone formation and, indirectly, bone resorption by osteoclasts (1). PTH also targets bone marrow stromal cells (BMSCs), favoring their differentiation in the osteoblast lineage at the expense of the adipocyte lineage (2). Whereas prolonged elevation of circulating levels of PTH, as in hyperparathyroidism, increases turnover and leads to bone loss, intermittent daily PTH increases bone mass and is used as an anabolic agent in the treatment of osteoporosis (3–6).

In target cells, PTH acts by binding to the PTH/PTH-related peptide (PTHrP) receptor (PTH1R), a G protein–coupled receptor (GPCR) (7). PTH activates primarily Gs and Gq, which in turn regulate the activity of adenylyl cyclase and phospholipase C (PLC) that control the flux of cAMP/protein kinase A (PKA) and protein kinase C (PKC)/Ca^2+^ signaling cascades, respectively (8). Mechanisms currently understood that lead to the regulation of bone formation and resorption downstream of the PTH1R comprise mainly the action of cAMP and PKA, which, in osteocytes, inhibit the activity of SIK2 and -3, allowing the nuclear translocation on the one hand of HDAC4/5 to repress Mef2C and decrease Sost expression, favoring bone formation, and on the other of CRTC2 to activate CREB and the expression of RANKL, increasing bone resorption (9, 10). Similarly, the PKA cascade has been shown to affect cell fate decision between osteoblasts and adipocytes (2, 11). The potential effects of the activation of the PKC and PLC cascades in the regulation of BMSCs and bone cells remain, however, poorly characterized.

PTH1R signaling is not the only signaling pathway that exerts an influence on bone turnover and homeostasis and on BMSCs. Crosstalk between PTH/PTHrP and WNT, apart from the regulation of Sost, or with TGF-β signaling, for instance, has been reported (10, 11), resulting in synergistic (12) or antagonistic effects (13). In contrast, although the Hippo pathway plays a crucial role in the regulation of several biological processes and in bone development, BMSC differentiation, and cell fate decision between adipogenesis and osteogenesis (14–16), whether PTH/PTHrP and Hippo signaling interact with each other has not been explored.

An unusual characteristic of the Hippo pathway is that neither the receptor(s) nor the ligand(s) that lead to its activation or inhibition have been identified. It can be regulated by some GPCRs (17–19), but whether the PTH1R and its ligands (PTH and PTHrP) regulate Hippo signaling is not known. Furthermore, the Hippo pathway, and in particular YAP and TAZ, are essential components of the mechanosensing machinery in stromal and bone cells (20, 21), and PTH signaling synergizes with mechanical loading in exerting its anabolic effects on bone (9). Last, both PTH (2) and Hippo (22) signaling have been shown to influence the cell fate decision between bone and fat.

Thus, whether PTH1R is one of the GPCRs that regulate Hippo signaling is a very relevant question. When Hippo signaling is on, YAP and TAZ undergo serine phosphorylation on specific residues, decreasing their stability, nuclear translocation, and transcriptional activity (23, 24). When Hippo signaling is off, YAP and TAZ are not serine-phosphorylated, accumulate in the cytoplasm, and translocate to the nucleus to promote target gene expression (25–30). Although it is recognized that YAP and TAZ contribute to bone homeostasis (31), they have been reported to either promote or inhibit osteoblast differentiation in vivo, according to the stage of osteoblast differentiation: Deletion in mesenchymal cells and osteoblast progenitors leads to increased bone mass, but an opposite effect is observed when YAP is deleted in mature osteoblasts and osteocytes (32). It is therefore unclear how YAP and/or TAZ transcriptional activity affects BMSCs, osteoblasts, and skeletal homeostasis.

Here, bulk RNA sequencing (RNA-Seq) of a murine BMSC cell line (W-20) treated with PTH revealed significant changes in Hippo signaling and YAP target gene expression. We therefore investigated the potential role of PTH in the regulation of YAP in this BMSC line and in osteocyte cell lines (Ocy454 and omGFP66) and explored the mechanisms by which the Hippo pathway was affected and the effects of this crosstalk on BMSCs. We report that PTH stabilizes YAP by decreasing YAP/LArge Tumor Suppressor kinase 1 (LATS1) interaction and YAP^S127^ phosphorylation via a PLC/Ca^2+^-dependent increase in Src kinase activity, resulting in YAP tyrosine phosphorylation. We identified Y428 in an Src phosphorylation consensus site on YAP as a key residue phosphorylated by Src in response to PTH and showed that preventing its phosphorylation leads to pronounced YAP instability, blocking BMSCs’ osteogenic and adipogenic differentiation. Mimicking YAP^Y428^ phosphorylation led to effects similar to PTH on YAP and BMSC differentiation. Together, these results demonstrate active crosstalk between the PTH/PTHrP and the Hippo signaling pathways and reveal that PTH signaling utilizes PLC and Src to counteract Hippo signaling and favor the differentiation of BMSCs.

## Results

### The Hippo signaling pathway is a target of PTH1R signaling in the W-20 BMSC cell line.

**Figure 1:**
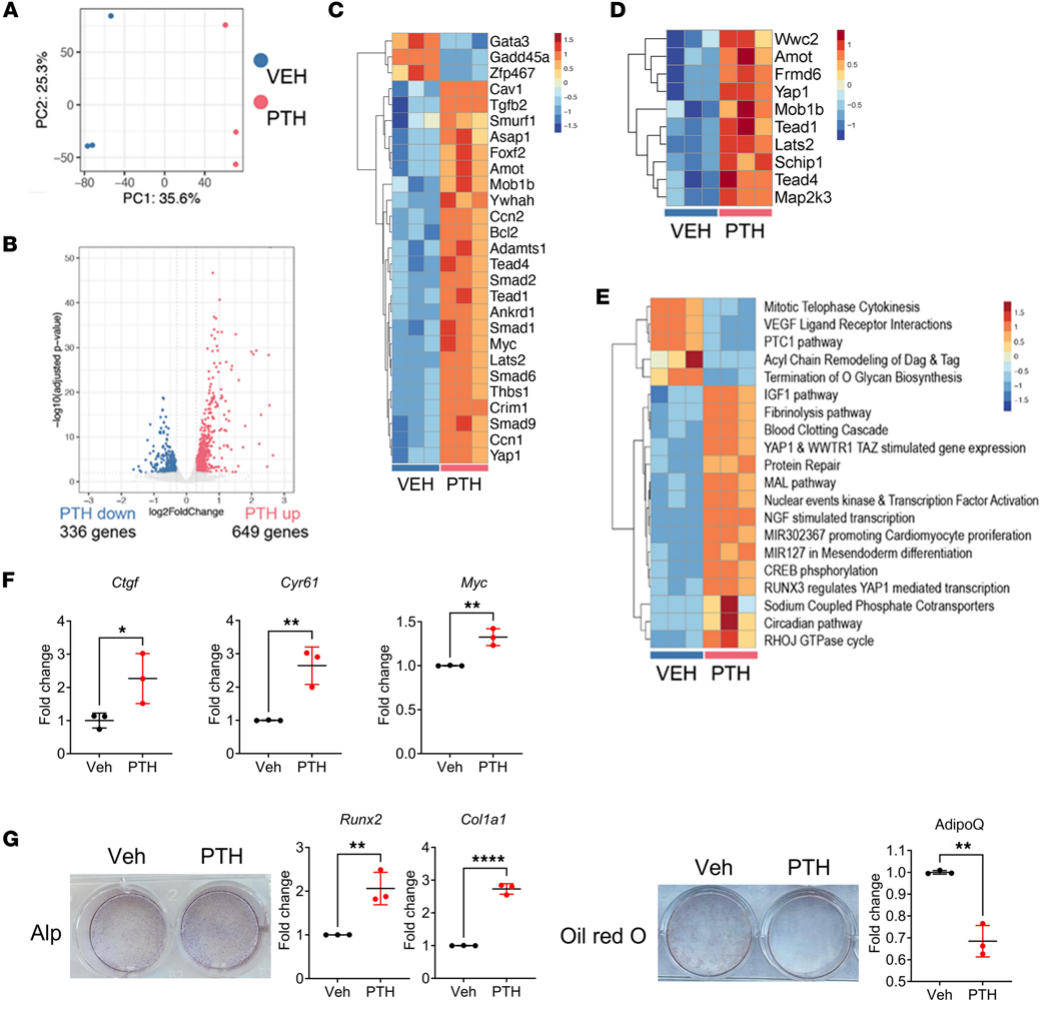
PTH regulates the Hippo pathway in W-20 cells. (**A**) Visualization of bulk RNA-Seq data with PCA plot of gene expressions. (**B**) Volcano plot of differentially regulated genes. Genes significantly upregulated and downregulated by PTH are in salmon and blue, respectively. (**C**) Heatmap of significantly differentially regulated YAP-related genes and (**D**) Hippo signal–related genes. (**E**) Heatmap of significantly up- or downregulated pathway terms by PTH stimulation. (**F**) Expression of selected YAP target genes with or without PTH treatment after OB differentiation. (**G**) Representative image of Alp and Oil Red O staining and expression of selected OB and Adi markers after OB- or Adi-induced differentiation with or without PTH treatment. Data are shown as the mean ± SEM of 3 independent experiments. *P < 0.05, **P < 0.005, ****P < 0.0001 by unpaired Student’s *t* test. The fold change is relative to the vehicle (Veh). Panel **F** mean Ct values: Gene *Ctgf* Veh 29.96; PTH 27.84. Gene *Cyr61* Veh 22.65; PTH 21.31. Gene *Myc* Veh 27.93; PTH 27.38. Panel **G** mean Ct values: Gene *Runx2* Veh 27.70; PTH 25.75. Gene *Col1a1* Veh 27.60; PTH 25.45. Gene *AdipoQ* Veh 16.96; PTH 20.22.

To assess the impact of PTH on the transcriptome of W-20 BMSCs, focusing on signaling pathways other than the classical cAMP pathway, we performed bulk RNA-Seq of W-20 BMSCs after 2 hours of exposure to PTH or to vehicle. As shown in Figure 1A, the principal component analysis (PCA) showed high similarities between each group of samples (PTH-treated versus vehicle-treated). Differential gene expression analysis identified 649 genes significantly upregulated by PTH, whereas 336 genes were significantly downregulated (Figure 1B). Strikingly, among the differentially expressed genes, YAP, YAP-related genes (Figure 1C), and Hippo signal–related genes (Figure 1D) were significantly altered by PTH treatment. For the most part, the effect of PTH was to increase the expression of YAP and of many YAP target genes as well as Hippo signaling–related genes. Pathway analysis also showed that YAP-stimulated gene expression was highly upregulated after PTH treatment (Figure 1E).

To confirm that PTH increased YAP and YAP target gene expression in W-20 cells, we differentiated W-20 cells into osteoblasts (OBs) in the presence of intermittent PTH (50 nM, 2 h/d for 4 days). PTH treatment increased significantly the expression of the YAP target genes *Ctgf*, *Cyr61*, and *Myc*, verifying that PTH increased YAP transcriptional activity during OB differentiation of W-20 cells (Figure 1F). As shown in Figure 1G, and as expected, PTH also favored OB differentiation in W-20 cells, as shown by an increase in alkaline phosphatase (Alp) staining and *Runx2* and *Col1a1* expression, with decreased adipocyte (Adi) differentiation, shown by a decrease in Oil Red O staining and expression of *Adiponectin* (*AdipoQ*) (Figure 1G).

These results suggested that activation of PTH1R signaling by PTH in W-20 BMSCs not only favors OB and represses Adi differentiation but also activates the transcription of YAP target genes, effectively turning Hippo signaling off.

### PTH promotes YAP stability in W-20 cells and OCY454 and OmGFP66 osteocytes by decreasing YAP^S127^ phosphorylation levels and increasing YAP target gene expression.

**Figure 2:**
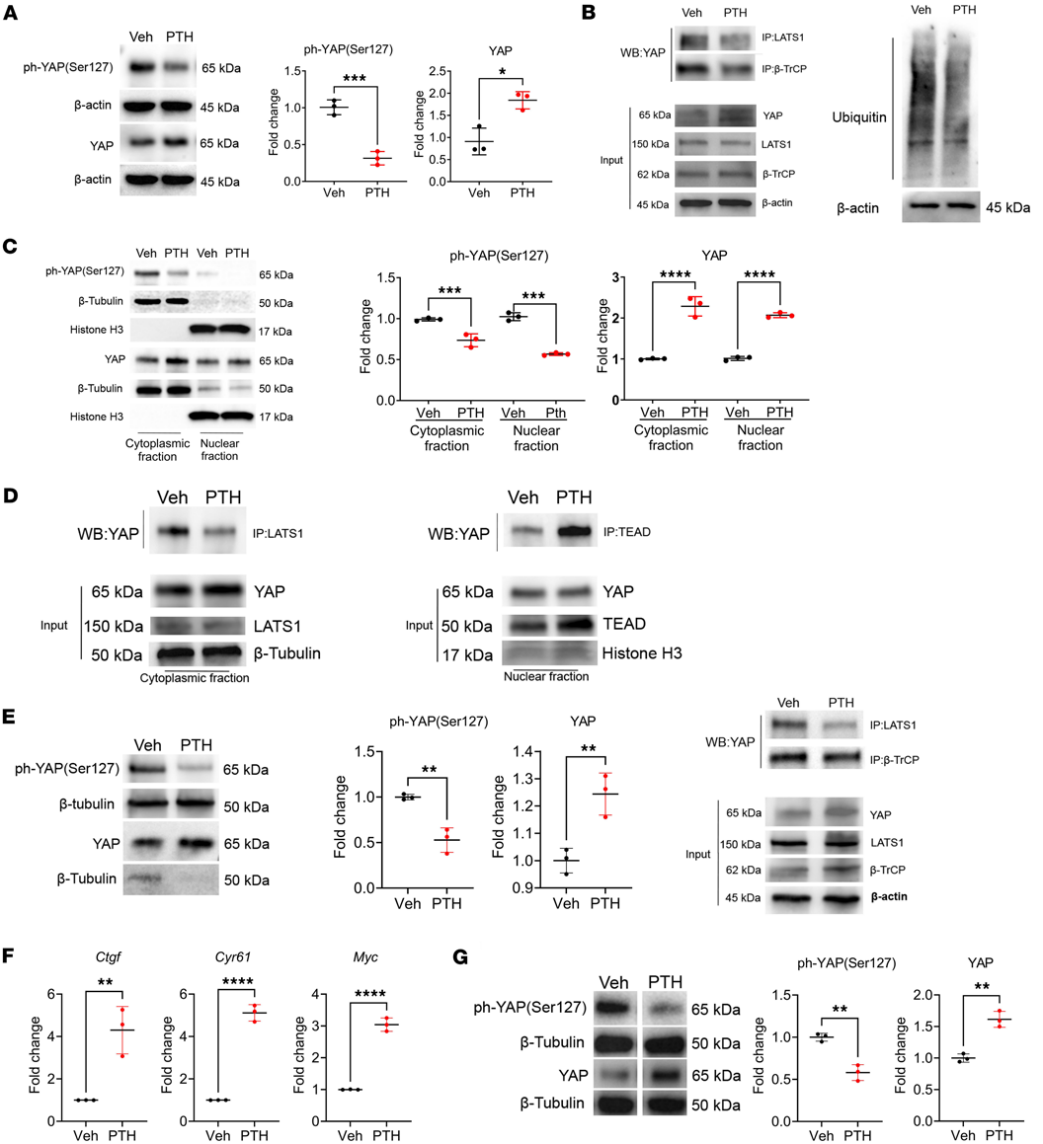
PTH promotes YAP stability in W-20 cells and in OCY454 and OmGFP66. (**A**) Western analysis representative blots and quantification of ph-YAP(S127) and YAP with or without PTH treatment. Fold change is relative to Veh. Protein levels were normalized to the respective housekeeping proteins in the same sample. (**B**) Representative blots of co-IP of YAP with LATS1 and β-TrCP and ubiquitin levels with or without PTH treatment. (**C**) Western analysis representative blots and quantification of ph-YAP (S127) and YAP protein levels in the cytoplasmic and nuclear fractions with or without PTH treatment. Fold change is relative to Veh. Protein levels were normalized to the respective housekeeping proteins in the same fraction/sample. (**D**) Representative blots of co-IP of YAP with LATS1 in the cytoplasmic fraction and TEAD in the nuclear fraction. (**E**) Western analysis representative blots and quantification of ph-YAP(S127) and YAP and representative blots of co-IP of YAP with LATS1 and β-TrCP with or without PTH treatment in OCY454 cells. (**F**) Expression of selected YAP target genes with or without PTH treatment in OCY454 cells. (**G**) Western analysis representative blots and quantification of ph-YAP(S127) and YAP with or without PTH in OmGFP66 cells. Data are shown as the mean ± SEM of 3 independent experiments. *P < 0.05, **P < 0.005, ***P < 0.0005, ****P < 0.0001 by unpaired Student’s *t* test. The fold change is relative to the Veh. Panel **F** mean Ct values: Gene *Ctgf* Veh 24.7; PTH 22.78. Gene *Cyr61* Veh 23.17; PTH 20.4. Gene *Myc* Veh 25.51; PTH 22.8.

Transcriptional events downstream of YAP are mostly the results of its stabilization in the cytosol and nuclear translocation. Given that YAP and its target genes were upregulated by PTH treatment in bulk RNA-Seq analysis (Figure 1), we assessed the effect of PTH on YAP stability. For this purpose, we stimulated W-20 cells with PTH (50 nM) for 2 hours and analyzed the phosphorylation level of YAP^S127^, a serine residue that when phosphorylated favors YAP degradation (33–35). PTH treatment significantly decreased the phosphorylation level of YAP^S127^ whereas the total protein level of YAP was significantly increased, indicating that PTH prevents YAP^S127^ phosphorylation, promoting YAP stability (Figure 2A).

Phosphorylation of YAP at S127 is the result of the interaction between YAP and the serine kinase LATS1. We therefore determined whether this protein-protein interaction was also altered by PTH treatment. Co-immunoprecipitation analysis showed that PTH decreased the interaction between YAP and LATS1, explaining the decrease in YAP^S127^ phosphorylation, which prevents the recruitment of the ubiquitin ligase β-TrCP and, thereby, YAP ubiquitination and degradation (Figure 2B). Further evidence for PTH-induced stabilization of YAP was provided by the finding that YAP nuclear translocation was also increased. As shown in Figure 2C, upon PTH treatment, YAP^S127^ phosphorylation levels were decreased in the cytoplasmic and in the nuclear fraction, while the total YAP was significantly increased in both the cytoplasm and nuclear fractions (Figure 2C). In addition, immunoprecipitation analysis showed that upon PTH stimulation, YAP interaction with LATS1 was decreased in the cytoplasmic fraction, while YAP interaction with its binding partner TEA domain transcription factor 1 (TEAD) in the nucleus was markedly increased (Figure 2D), explaining the increase in the expression of YAP target genes, such as *Ctgf*, *Cyr61*, and *Myc*, compared with vehicle-treated cells.

Similar findings on the effect of PTH on YAP stability were also seen in the osteocyte cell lines OCY454 (Figure 2, E and F) and OmGFP66 (Figure 2G), establishing the fact that this regulatory mechanism also occurs in other bone cells known to express the PTH1R and respond to PTH.

### PTH-induced YAP stabilization occurs via the PLC/Ca^2+^/Src axis.

**Figure 3:**
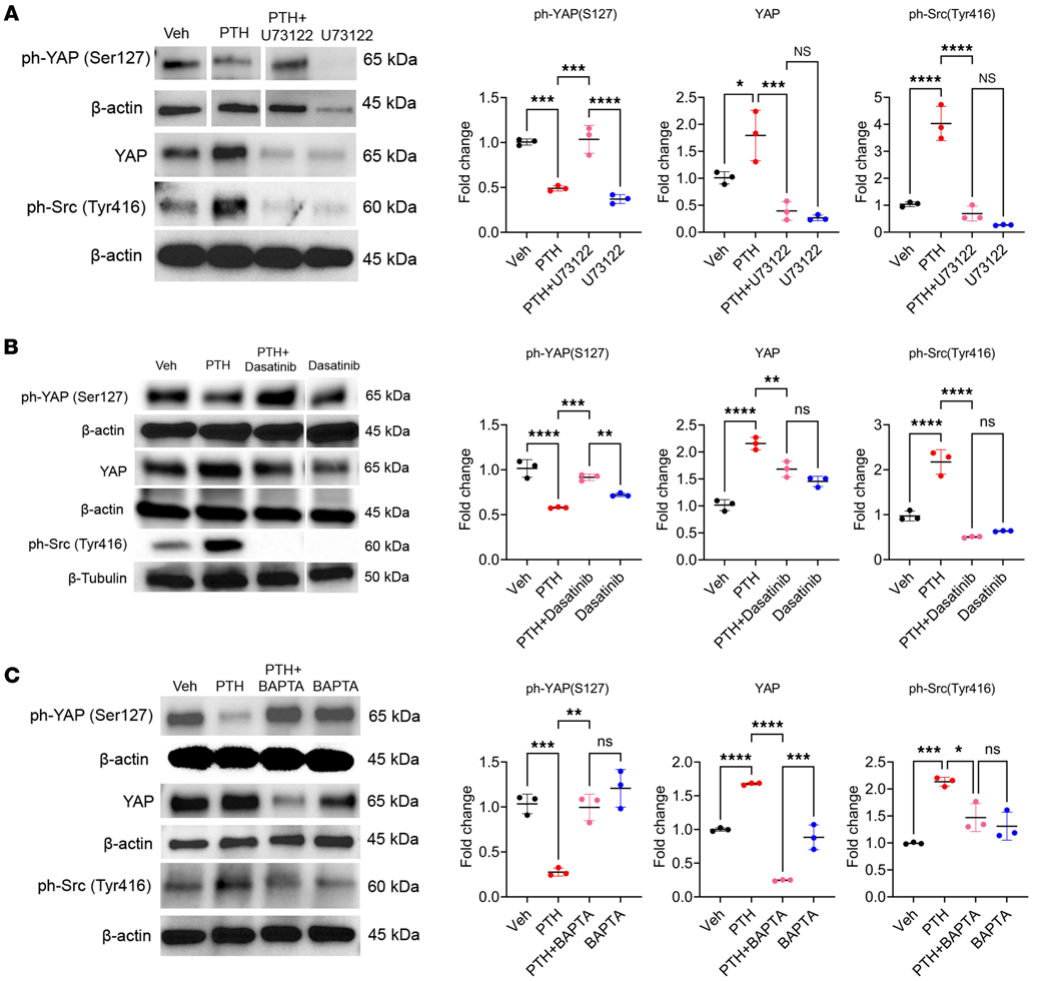
PTH stabilizes YAP via the PLC/Ca^2+^/Src axis. (**A**) Western analysis representative blots and quantification of ph-YAP(S127), YAP, and ph-Src(Y416) with or without PTH and U73122 treatment or the combination of the two. (**B**) Western analysis representative blots and quantification of ph-YAP(S127), YAP, and ph-Src(Y416) with or without PTH and dasatinib treatment or the combination of the two. (**C**) Western analysis representative blots and quantification of ph-YAP(S127), YAP, and ph-Src(Y416) with or without PTH and BAPTA treatment or the combination of the two. Data are shown as the mean ± SEM of 3 independent experiments. *P < 0.05, **P < 0.005, ***P < 0.0005, ****P < 0.0001 by 1-way ANOVA followed by Tukey’s test for multiple comparisons. The fold change is relative to the Veh.

To determine whether the effect of PTH on YAP^S127^ phosphorylation and YAP stabilization involved the PKA or the PKC pathways, we cotreated the cells with PTH and the PKA-specific inhibitor H89 or the PKC-specific inhibitor G06983 (36, 37). Blocking either of these pathways failed to prevent the PTH-dependent decrease in YAP^S127^ phosphorylation (Supplemental Figure 1; supplemental material available online with this article; https://doi.org/10.1172/jci.insight.191245DS1). PTH also activates PLC to mobilize intracellular Ca^2+^ (38). We found that cotreatment of cells with PTH and the PLC-specific inhibitor U73122 (39) prevented the PTH-induced decrease in YAP^S127^ phosphorylation (Figure 3A). As shown in Figure 3, A–C, PTH also activated Src kinase activity, as indicated by increased Src^Y416^ phosphorylation, and this srcTyr416 phosphorylation was blocked by U73122. Cotreatment with PTH and the specific Src family kinase inhibitor dasatinib (40) blocked PTH-induced YAP stabilization, as shown by a marked increase in YAP^S127^ phosphorylation and decreased total YAP protein levels (Figure 3B). Importantly, chelation of intracellular Ca^2+^ with BAPTA suppressed PTH-dependent Src^Y416^ phosphorylation and increased YAP^S127^ phosphorylation in response to PTH (Figure 3C).

These results demonstrated that PTH regulates YAP stability via the PLC/Ca^2+^/ Src kinase cascade.

### Identification of tyrosine 428 as the target of PTH-induced Src-dependent stabilization of YAP.

**Figure 4:**
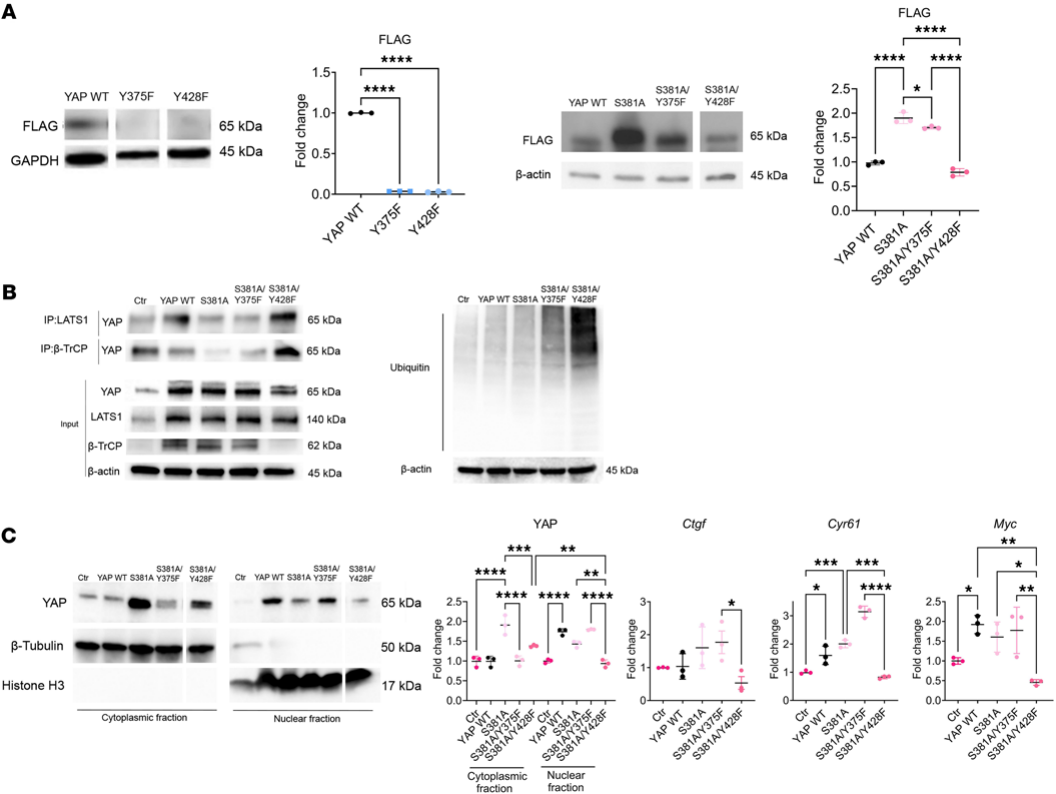
YAP^Y428^ mimics the effect of PTH-induced Src-dependent stabilization of YAP. (**A**) Western analysis representative blots and quantification of YAP WT and mutants (using a FLAG antibody) in W-20 WT, W-20^Y381F^, W-20^Y428F^, W-20^S381A/375F^, or W-20^S381A/Y428F^ cells. (**B**) Representative blots of co -IP in W-20 (control) and W-20 WT, W-20^S381A/375F^, or W-20^S381A/Y428F^, using LATS1 and β-TrCP antibodies, and of ubiquitin levels. (**C**) Western analysis representative blots and quantification of YAP protein levels in W-20 WT, W-20^S381A/375F^, or W-20^S381A/Y428F^, in the cytoplasmic and nuclear fraction and the expression of selected YAP target genes. Data are shown as the mean ± SEM of 3 independent experiments. *P < 0.05, **P < 0.005, ***P < 0.0005, ****P < 0.0001 by 1-way ANOVA followed by Tukey’s test for multiple comparisons. Ctr, nontransfected W-20 cells; YAP WT, W-20 overexpressing the WT YAP. The fold change for panel **A** is relative to WT, and the fold change for panels **B** and **C** is relative to Ctr. Panel **C** mean Ct values: Gene *Ctgf* Ctr 26.36; YAP WT 25.07911; S381A 25.07442; S381A/Y375F 23.95; S381A/Y428F 23.95. Gene *Cyr61* Ctr 27.99; YAP WT 26.88; S381A 26.62; S381A/Y375F 28.34; S381A/Y428F 28.96. Gene *Myc* Ctr 25.96; YAP WT 25.08; S381A 24.62; S381A/Y375F 26.60; S381A/Y428F 28.17.

Given our finding of Src’s role in the regulation of YAP stability by PTH, we searched for consensus sequences for Src-dependent tyrosine phosphorylation sites on YAP. We identified 2 Src consensus sequences, one including YAP^Y375^ (PFLNSGTYHSRDEST) and the other including YAP^Y428^ (SQQSRFPDYLEA). To determine the potential role of YAP^Y375^ and YAP^Y428^ phosphorylation in YAP stabilization and function by PTH, we mutated these tyrosines to phenylalanine to prevent phosphorylation (Y375F and Y428F) and generated mutated W-20 stable cell lines (YAP^Y375F^ or YAP^Y428F^ W-20 cells). Western blot analysis of YAP^Y375F^ or YAP^Y428F^ cell lysates showed that both mutations led to a significant decrease in YAP protein levels, demonstrating a crucial role for these 2 tyrosines in the regulation of YAP stability (Figure 4A). The destabilization of YAP was, however, so pronounced as to prevent further analysis of changes in the protein and its interactions. To prevent excessive YAP destabilization, we generated W-20 stable cell lines with YAP^S381^ mutated into alanine (YAP^S381A^), in addition to Y375F or Y428F (YAP^S381A/Y375F^ and YAP^S381A/Y428F^ W-20 cells). YAP^S381^ is known to be phosphorylated by LATS1, leading to the recruitment of the β-TrCP ubiquitin ligase and ultimately favoring YAP degradation (33). Thus, as expected, YAP^S381A^ mutation stabilized both YAP^Y375F^ and YAP^Y428F^ protein levels and allowed us to detect YAP in both mutant cell lines. However, whereas introducing the YAP^381A^ mutation fully stabilized the YAP^S381A/Y375F^ mutant protein, the YAP^S381A/Y428F^ mutant protein was only partially stabilized compared with YAP^S381A^ and YAP ^S381A/Y375F^ (Figure 4A). Similarly, while YAP^S381A/Y375F^ mutation did not alter the level of interaction of YAP with LATS1 and β-TrCP compared with YAP^S381A^ mutation alone, YAP^S381A/Y428F^ mutation enhanced the interaction of YAP with LATS1 and β-TrCP, resulting in increased YAP ubiquitination compared with both YAP^S381A^ and YAP^S381A/Y375F^ mutations (Figure 4B). In addition, YAP^S381A/Y428F^ mutation retained YAP in the cytosol, impairing induction of YAP target gene expression (*Ctgf*, *Cyr61*, and *Myc*) compared with YAP^S381A^ and YAP^S381A/Y375F^ mutations (Figure 4C).

These results established that YAP^Y428^ plays a crucial role in regulating YAP stability.

### Mutating YAP^Y428^ to the phosphomimetic aspartic acid (YAP^Y428D^) mimics PTH effects on YAP phosphorylation and stability.

**Figure 5:**
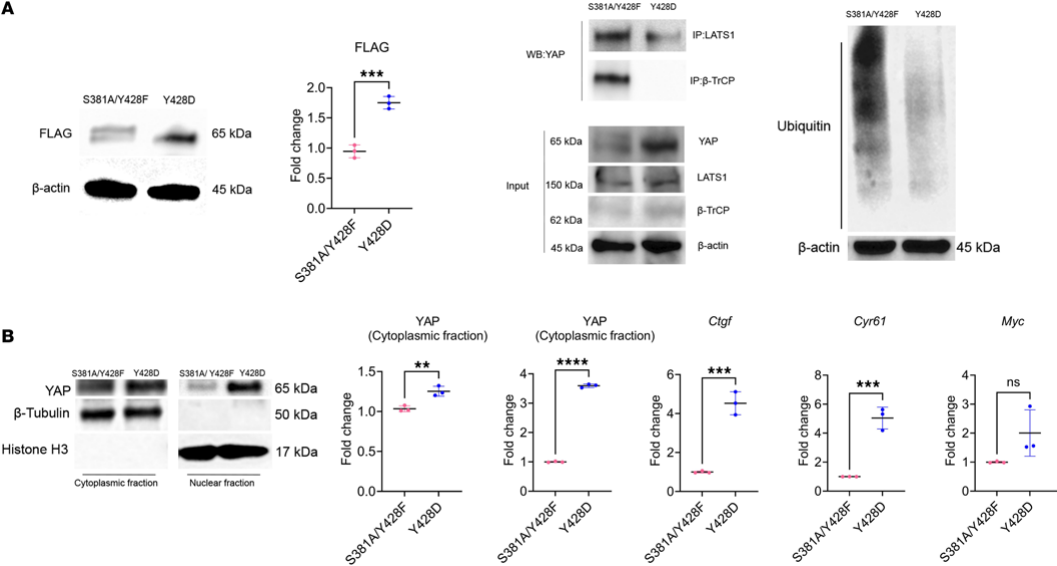
The phosphomimetic YAP^Y428D^ mirrors the effect of PTH on YAP phosphorylation and stability. (**A**) Representative blots and quantification by Western analysis of YAP level in W-20^S381A/Y428F^ and W-20^Y428D^ cells using a FLAG antibody, immunoprecipitation using LATS1 and β-TrCP antibodies, and ubiquitin level. (**B**) Representative blots and quantification of YAP level in W-20^S381A/Y428F^ and W-20^Y428D^ in the cytoplasmic and nuclear fractions and the expression of selected YAP target genes. Data are shown as the mean ± SEM of 3 independent experiments. **P < 0.005, ***P < 0.0005, ****P < 0.0001 by unpaired Student’s *t* test. The fold change is relative to S381A/Y428F. Panel **B** mean Ct values: Gene *Ctgf* S381A/Y428F 27.88; Y428D 24.77. Gene *Cyr6*1 S381A/Y428F 27.50; Y428D 35.38. Gene *Myc* S381A/Y428F 26.32; Y428D 32.77.

According to our findings, mimicking YAP^Y428^ phosphorylation should exert the same influence on YAP and W-20 cells as PTH treatment. To verify the role of YAP^Y428^ phosphorylation in YAP stability, we generated a W-20 stable cell line with a phosphomimetic Y428 mutation by substituting the tyrosine into aspartic acid (YAP^Y428D^) (Figure 5A). YAP^Y428D^ mutation stabilized YAP on its own, as shown by an increased YAP protein level and decreased interaction with LATS1 and β-TrCP, decreasing ubiquitination when compared with YAP^S381A/Y428F^ (Figure 5A). YAP^Y428D^ mutation also allowed for higher YAP protein levels in the nucleus and consequent increase in the expression of the YAP target genes *Ctgf*, *Cyr61*, and *Myc* (Figure 5B). Thus, mimicking the phosphorylation of YAP^Y428^ by substitution to aspartic acid mimicked closely the response of W-20 cells to PTH treatment.

### The phosphorylation of YAP^Y428^ is required for PTH-induced YAP stabilization.

**Figure 6:**
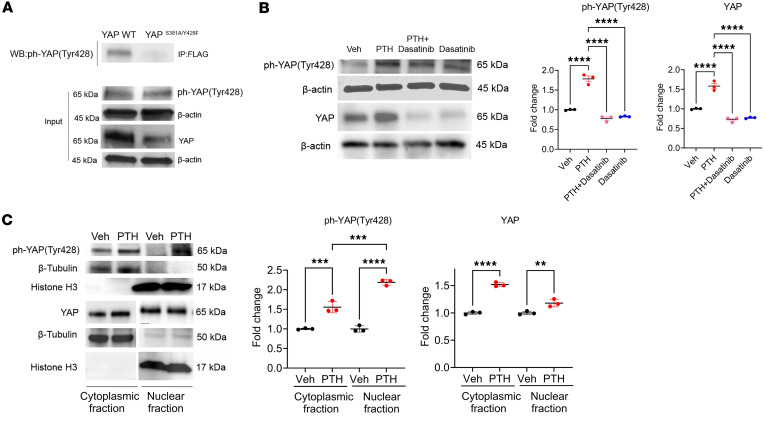
PTH phosphorylates YAP^Y428^ via Src. (**A**) Representative blots of co-IP of YAP(Tyr428) in W-20WT or W-20^S381A/Y428F^ using FLAG antibody. (**B**) Western analysis representative blots and quantification of ph-YAP(Tyr428) and YAP protein levels with or without PTH, dasatinib treatment, or the combination of the two. (**C**) Western analysis representative blots and quantification of ph-YAP(Tyr428) and YAP protein levels in the cytoplasmic and nuclear fractions with or without PTH treatment. Data are shown as the mean ± SEM of 3 independent experiments. **P < 0.005, ***P < 0.0005, ****P < 0.0001 by 1-way ANOVA followed by Tukey’s test for multiple comparisons. The fold change is relative to the Veh.

To determine whether PTH-induced stabilization of YAP involves YAP^Y428^ phosphorylation, we developed an antibody specific for phosphorylated YAP^Y428^. As shown in Figure 6A, the antibody detected basal phosphorylated YAP^Y428^ but not YAP^Y428F^, and the phosphorylated band was extinguished by treatment with dasatinib in the presence of PTH (Figure 6B), establishing its specificity for both Y428 and its phosphorylated state. We then used this antibody to determine whether PTH induced the phosphorylation of YAP^Y428^ to increase YAP protein stability. As shown in Figure 6B, YAP^Y428^ was indeed phosphorylated in response to PTH. This event required the activity of Src as cotreatment with the Src inhibitor dasatinib blocked the PTH-induced increase in YAP^Y428^ phosphorylation and the increase in YAP protein levels (Figure 6B). Furthermore, phosphorylated YAP^Y428^ was enriched in the nuclear fraction after PTH treatment (Figure 6C).

**Figure 7:**
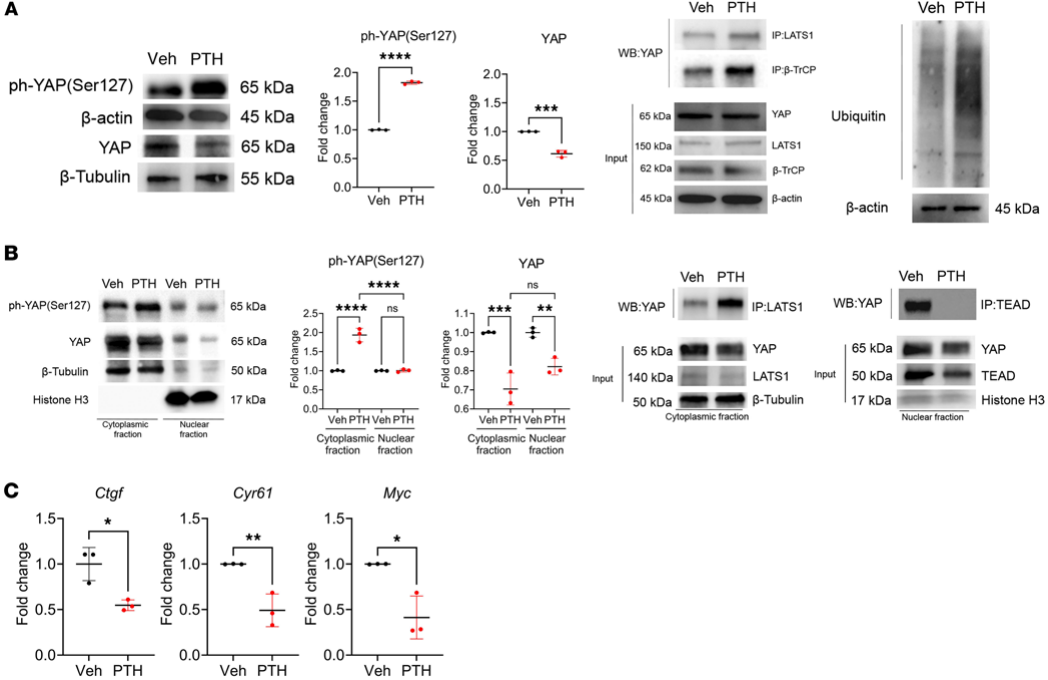
YAP^Y428^ phosphorylation is required for PTH-induced YAP stabilization. (**A**) Western analysis representative blots and quantification of ph-YAP(S127) and YAP protein levels in W-20^S381A/Y428F^ cells with or without PTH treatment; representative blots of immunoprecipitation in W-20^S381A/Y428F^ cells using LATS1 and β-TrCP antibodies and ubiquitin levels with or without PTH treatment. Data are shown as the mean ± SEM of 3 independent experiments. ***P < 0.0005, ****P < 0.0001 by unpaired Student’s *t* test. (**B**) Western analysis representative blots and quantification of ph-YAP(S127) and YAP protein levels in the cytoplasmic and nuclear fractions of W-20^S381A/Y428F^ cells treated with or without PTH and immunoprecipitation using LATS1 antibody in the cytoplasmic fraction and TEAD antibody in the nuclear fraction of W-20^S381A/Y428F^ cells. Data are shown as the mean ± SEM of 3 independent experiments. **P < 0.005, ***P < 0.0005, ****P < 0.0001 by 1-way ANOVA followed by Tukey’s test for multiple comparisons. (**C**) Expression of selected YAP target genes in W-20S^381A/Y428F^ cells treated with or without PTH. Data are shown as the mean ± SEM of 3 independent experiments. *P < 0.05, **P < 0.005, by unpaired Student’s *t* test. The fold change is relative to the Veh. Panel **B** mean Ct values: Gene *Ctgf* Veh 24.016; PTH 25.18. Gene *Cyr61* Veh 17.76; PTH 19.12. Gene *Myc* Veh 23.94; PTH 25.65.

Having established that PTH induced YAP^Y428^ phosphorylation, we tested whether preventing this event by substituting a phenylalanine to the tyrosine altered PTH effects on YAP in W-20 cells. In sharp contrast with its effects in WT cells, in cells expressing YAP^S381A/Y428F^, PTH treatment increased YAP^S127^ phosphorylation and decreased YAP protein levels, with an increase in LATS1 and β-TrCP interaction, shown by co-immunoprecipitation, and in YAP ubiquitination (Figure 7A). These changes had the expected consequences on the nuclear translocation of YAP (Figure 7B) and on target gene expression (Figure 7C), which were markedly decreased. Since the W-20 cell line used in these studies contains both the YAP^S381A^ and the YAP^Y428F^ mutations, we treated YAP^S381A^ mutant cells with PTH to determine if the effect of PTH on YAP stability observed in Figure 6 was specifically mediated by YAP^Y428^ mutation, excluding any potential effect of the YAP^S381A^ mutation. PTH treatment of YAP^S381A^ mutant cells mimicked all the effects of PTH in WT W-20 cells, verifying that the observed effect of PTH on YAP stability was specifically mediated by YAP^Y428^ phosphorylation (Supplemental Figure 2). Finally, we also determined whether preventing YAP^Y375^ phosphorylation had the same effects as YAP^Y428^, using the YAP^S381A/Y375F^ mutant cells. Preventing the phosphorylation of YAP^Y375^ had no measurable effects on YAP response to PTH (Supplemental Figure 3). These results are in agreement with the observation that total YAP protein levels remained stable in the YAP^381A/375F^ cells (Figure 4). Together these results established that YAP^Y428^ phosphorylation plays a specific and unique role in the regulation of YAP stability and transcriptional activity by PTH.

### Role of YAP^Y428^ phosphorylation and YAP in W-20 differentiation into OBs and Adi.

**Figure 8:**
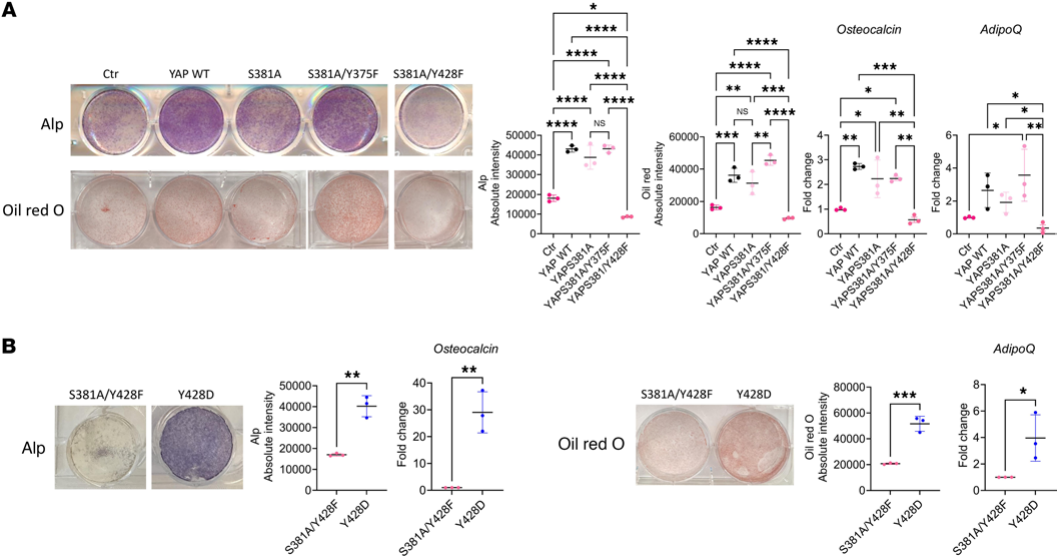
YAP^Y428^ phosphorylation status regulates W-20 differentiation into OB and Adi. (**A**) Representative images of Alp staining, Oil Red O staining, and expression of *Osteocalcin* and *AdipoQ* after OB- and Adi-induced differentiation, in W-20 Ctr, W-20WT, W-20^S381A^, W-20^S381A/375F^, W-20S^381A/Y428F^, and W-20^Y428D^ cells. Data are shown as the mean ± SEM of 3 independent experiments. *P < 0.05, **P < 0.005, ***P < 0.0005, ****P < 0.0001 by 1-way ANOVA followed by Tukey’s test for multiple comparisons (**B**) Representative image of Alp and Oil Red O staining and expression of *Osteocalcin* and *AdipoQ* after OB- and Adi-induced differentiation, in W-20^S381A/Y428F^ and W-2^0Y428D^ cells. Data are shown as the mean ± SEM of 3 independent experiments. *P < 0.05, **P < 0.005, ***P < 0.0005 by unpaired Student’s *t* test. The fold change for panel **A** is relative to the Ctr, and the fold change for panel **B** is relative to S381A/Y428F. Panel **A** mean Ct values: Gene *Osteocalcin* Ctr 27.43; YAP WT 26.18; S381A 26.91; S381A/Y375F 26.45; S381A/Y428F 28.07. Gene *AdipoQ* Ctr 21.73; YAP WT 18.57; S381A 18.96; S381A/Y375F 18.58; S381A/Y428F 21.47. Panel **B** mean Ct values: Gene *Osteocalcin* S381A/Y428F 33.44; Y428D 34.12. Gene *AdipoQ* *S*381A/Y428F 30.27; Y428D 23.08.

Given the ability of the W-20 cells to differentiate into either OBs or Adi, we then investigated the potential role of YAP^Y428^ in W-20 cell fate determination. All mutations had no significant effects on W-20 cell proliferation (Supplemental Table 1). In contrast, overexpression of WT YAP led to an increase in both OB differentiation, illustrated by Alp staining and *Osteocalcin* expression (Figure 8A), and Adi differentiation, illustrated by Oil Red O staining and *AdipoQ* expression (Figure 8A). Neither YAP^S381A^ nor YAP^S381A/Y375F^ mutant cells differed from WT YAP cells in their responses (Figure 8A). In contrast, YAP^S381A/Y428F^ mutation impaired W-20 cell differentiation along both the OB and the Adi lineage (Figure 8A), with significant decreases in *Osteocalcin* and *AdipoQ* relative to controls and WT YAP cells. These data suggest that YAP^Y428^ phosphorylation is required to promote W-20 cell differentiation into both lineages but is not involved in the cell fate decision toward OB or Adi. Supporting the fact that this is due to YAP^Y428^ phosphorylation, the YAP^Y428D^ mutation enhanced YAP functions, favoring both OB and Adi differentiation of YAP^Y428D^ cells as well as *Osteocalcin* and *AdipoQ* gene expression (Figure 8B), but it failed to mimic the PTH-mediated repression of the adipogenic lineage, suggesting that signaling events other than the PLC/Ca^2+^/Src cascade are required for PTH to repress adipogenesis. Taken together these data suggest that YAP^Y428^ is required for YAP stability and for a proper differentiation of BMSCs into both OB and Adi.

### Mutating YAP^Y428^ to phenylalanine (Y428F) alters the effects of PTH on W-20 cell differentiation, Hippo signaling, and transcriptomic profile.

**Figure 9:**
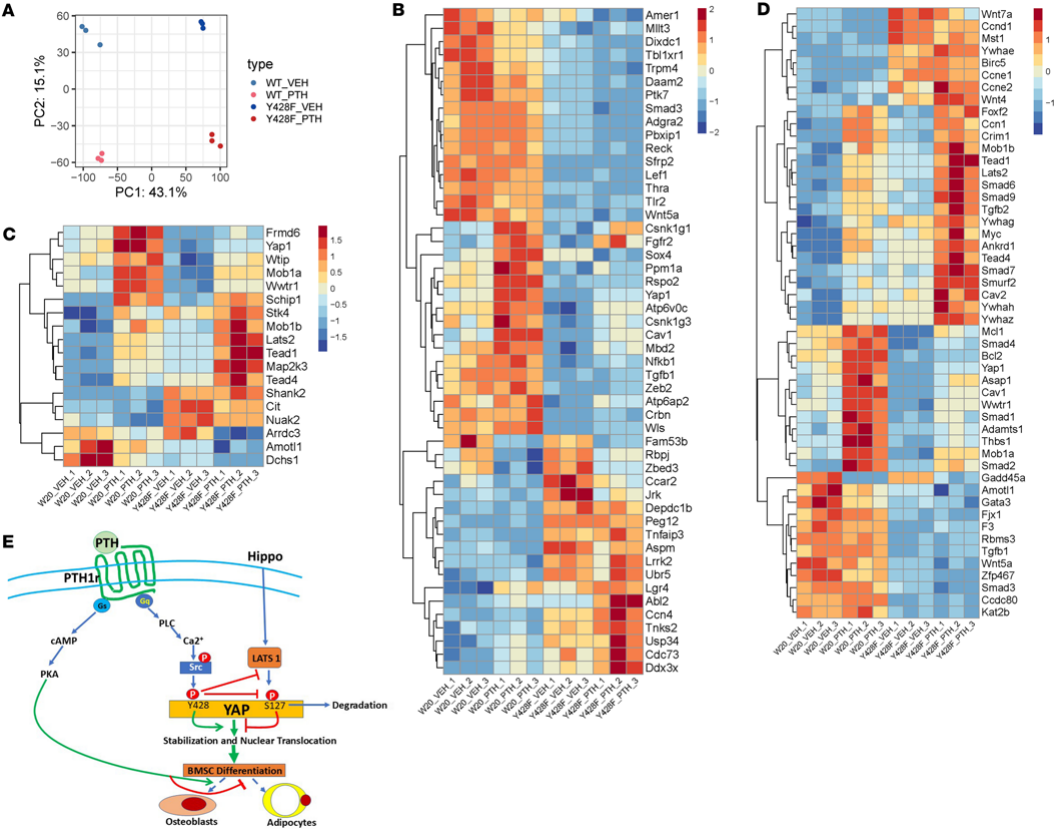
Y428F mutation alters the effects of PTH on W-20 cell transcriptomic profile. (**A**) Visualization of bulk RNA-Seq data with PCA plot of gene expressions. (**B**) Heatmap of top 50 significantly differentially regulated genes. (**C**) Heatmap of significantly differentially regulated Hippo signal–related genes. (**D**) Heatmap of significantly differentially regulated YAP-related genes. (**E**) Proposed mechanism of action: PTH regulates YAP through specific tyrosine residues, promoting its nuclear translocation and activation of target gene expression. This process occurs via the PLC/Ca^2+^/Src tyrosine kinase signaling cascade to influence YAP stability, antagonizing Hippo signaling and favoring stromal cell differentiation.

To further evaluate the importance of YAP^Y428^ phosphorylation in the response of W-20 cells to PTH, we then compared the cellular and transcriptomic responses of WT and YAP^S381A/Y428F^ W-20 cells to PTH. First, similar to the effect of YAP^S381A/Y428F^ mutation in unstimulated differentiation of cells (Figure 1), treatment of YAP^S381A/Y428F^ cells with PTH failed to induce their differentiation (Figure 8A). As shown in Figure 9, A–C, bulk RNA-Seq analysis of the genome-wide transcriptome of the treated cells revealed many significant differences between the responses of WT and YAP^S381A/Y428F^ cells, verifying that the ability of YAP^Y428^ to be phosphorylated is an essential component of the cellular response of BMSCs to PTH.

The bulk RNA-Seq results showed that a large number of genes were changed by the YAP^Y428F^ mutation, both in the absence of and in response to PTH. Figure 9A shows the PCA, whereas Figure 9B shows the top 50 genes (*P* < 0.0000001) altered by the YAP^Y428F^ mutation, which includes YAP, induced by PTH in control cells but not in mutated cells, and several components of the TGF-β/bone morphogenic protein (BMP) and WNT signaling pathways (*Smad3*, *Tgfb1*, *Sfrp2*, *Rspo2*, *Wnt5a*). We then focused our analysis on the significant (*P* < 0.0001) transcriptomic changes that occurred in Hippo signaling (Figure 9C) and YAP target genes (Figure 9D), to confirm the changes induced by PTH and altered by the YAP^Y428F^ mutation. Noticeably, the strong induction of *YAP* expression observed in WT cells was entirely blunted in YAP^S381A/Y428F^ cells, confirming that, in addition to inducing stabilization of the YAP protein, phosphorylation of YAP^Y428^ by PTH induces an increase in *YAP* transcription, suggesting a positive feedback loop. *YAP* was not the only gene the PTH response of which was blunted: The response of *Frmd6*, *Wtip*, *Mob1a*, and *Wwtr1*, which responded solidly to PTH, was lost in the YAP^S381A/Y428F^ cells. In contrast, another set of genes showed enhanced expression in response to PTH in YAP^S381A/Y428F^ cells: These included *Lats2*, *Tead1* and -*4*, *Mob1b*, and *Map2K3*. Further analysis revealed 4 highly relevant genes that were markedly increased in YAP^S381A/Y428F^ cells, independent of exposure to PTH: *Shank2*, *Cit*, *Nuak2*, and *Arrdc3*. Last, *Amotl1* and *Dchs1* were significantly decreased in YAP^S381A/Y428F^ cells. Most of these genes are well-established regulators of Hippo or YAP, and some have already been identified in osteogenesis (41–43). These results confirm the link between PTH1R and Hippo signaling and establish the importance of the PTH-induced phosphorylation of YAP^Y428^ in BMSCs’ differentiation and in the regulation of the expression of several components of Hippo signaling.

Because our results (Figure 8) showed clearly that the YAP^Y428F^ substitution also altered the differentiation of W-20 cells in both the OB and Adi lineages, we then analyzed changes in adipogenesis and osteogenesis gene sets. As shown in Supplemental Figure 4, A and B, mutating Y428 to F428 had a significant impact on these 2 gene sets and on their response to PTH. Many components of the TGF-β/BMP and of the WNT signaling pathways were affected (*Lrp5*, *Sfrp2*, *Rspo2*, *Fzd1*, *Tgfb1* and *-2*, several *Smad*s, *BMP4*, *Id1* and *-2*, and *BMPr1b*), including several WNT ligands. These findings verified that the ability of YAP^Y428^ to become phosphorylated is essential for BMSCs’ differentiation and for their response to PTH.

## Discussion

This report establishes for the first time to our knowledge a direct link between PTH signaling and the Hippo pathway. We show that PTH signaling regulates the Hippo pathway negatively in BMSCs and in osteocytes by increasing the expression and stability of YAP, a key target of Hippo signaling (Figure 9E). Mechanistically, we show in a BMSC cell line (W-20) that 1) downstream of the PTH1R, Src is activated via the PLC/Ca^2+^ pathway; 2) PTH-mediated Src activation leads to the phosphorylation of YAP^Y428^; 3) YAP^Y428^ is a key Src substrate, the phosphorylation of which results in YAP stabilization and increased YAP transcriptional activity, and 4) this tyrosine phosphorylation event is required for the differentiation of BMSCs in both the OB and Adi lineages, i.e., before the cell fate branching between these 2 lineages (Figure 9E). Thus, in addition to its known effects on PKA and cAMP in target cells (9), which we show here are not involved in YAP regulation, PTH also activates Src via the PLC pathway to turn the Hippo pathway off by increasing YAP expression, stability, and transcriptional activity, allowing stromal cell differentiation. Of note, a recent paper looking at integrating several databases exploring the effects of PTH on miRNAs in osteoblasts also noted the Hippo signaling pathway as one of the 3 most significant pathways affected by PTH (44).

This set of observations linking PTH and Hippo signaling is important because both pathways are known to modulate cell proliferation and differentiation, and both are involved in the OB and Adi differentiation of BMSCs (45–47). YAP and TAZ are transcriptional coactivators that are negatively regulated by the Hippo pathway: YAP is inhibited by the Hippo pathway kinase cascade, at least in part via phosphorylation of S127, which results in YAP 14–3–3 binding, cytoplasmic retention, and ubiquitin-dependent degradation (33). Thus, when YAP is stabilized, as shown here downstream of PTH1R signaling, it counteracts Hippo signaling.

Unlike traditional pathways that involve dedicated ligand-receptor pairing, a specific and dedicated extracellular ligand/receptor complex has not been identified for the Hippo pathway. However, a variety of signaling pathways, including mechanical cues, cell polarity, cell-cell adhesion, and hormones, can interact with and regulate Hippo to control cellular processes (48–50). Here, we identify the PTH1R as a GPCR that turns off Hippo signaling by stabilizing YAP and promoting YAP target gene expression.

The regulation of YAP by GPCRs has been observed for other receptors, and depending on which G subunit is involved, YAP can be either stabilized or degraded. For instance, serum-borne lysophosphatidic acid and sphingosine 1-phosphate act through G12/13-coupled receptors and inhibit LATS1/2, thereby stabilizing and activating YAP (19). In contrast, stimulation of Gs-coupled receptors by glucagon or epinephrine activates LATS1/2 kinase activity, thereby inhibiting YAP function (19), illustrating the complexity of the Hippo pathway regulation by GPCRs.

The receptor we activated here, PTH1R, couples to multiple heterotrimeric G proteins, including Gαi, Gαs, Gα q/11, and Gα12/13 (51, 52), activating both the PKA and the PLC/PKC pathways, and could therefore have the ability to stabilize or destabilize YAP. Although the activation of PLC by PTH signaling was well established, the role and the targets of this pathway have not, to our knowledge, been identified (53). Our results identify a specific role of PLC activation downstream of the PTH1R by showing that activation of the PLC/Ca^2+^/Src kinase axis by PTH regulates YAP^Y428^ phosphorylation and favors YAP stability. A link between Src and Hippo signaling has previously been reported (54–57) but not in the context of BMSCs or in relation with PTH signaling or YAP. The existence of this PTH signaling pathway is supported by our findings that inhibiting Src, PLC, or chelation of Ca^2+^ upon PTH exposure prevented the stabilization of YAP, and so did the substitution of Y428 by a phenylalanine. These findings are also consistent with previous findings showing that PTH can activate c-Src in enterocytes and kidney cells (58–60), but here again, no link was established with PLC signaling, with YAP stability, and/or with the differentiation of stromal cells.

Although we have focused on 2 previously identified Src tyrosine phosphorylation consensus sequences in YAP (61), which include YAP^Y375^ and YAP^Y428^, and we show that both affect YAP protein stability, YAP^Y428^ appears to be particularly important to stabilize YAP. Indeed, if nonphosphorylatable mutation of YAP^Y375^ or YAP^Y428^ destabilized YAP, but in YAP^Y428^ much more than in YAP^Y375^, only the mutation of YAP^Y428^ favored the interaction of YAP with LATS1, the recruitment of the ubiquitin ligase β-TrCP, and YAP ubiquitination, showing the specificity of YAP^Y428^’s role in YAP biology.

The role of tyrosine phosphorylation in YAP stability and of Src family kinases in this process has recently been reported in other cell types but was not related to any receptor. Whereas the phosphorylation of YAP on serine residues promotes its degradation (23), increasing evidence suggests that tyrosine phosphorylation has the opposite effect, favoring the stability of YAP (25, 62–65). For instance, Smoot et al. showed that activation of Src family kinases resulted in the phosphorylation of YAP on tyrosine 357 in cholangiocarcinoma downstream of PDGFR signaling (64). In the same cellular model, Src kinase inhibition resulted in loss of YAP^Y357^ phosphorylation and reduced YAP target gene expression (25).

The stabilization of YAP by PTH and the identification of the specific role of YAP^Y428^ phosphorylation in the regulation of YAP stability and in the response to PTH are potentially novel observations. We show that PTH phosphorylates YAP^Y428^ in an Src-dependent manner and blocking Src kinase activity not only reduced YAP^Y428^ phosphorylation but also prevented the stabilization of YAP in response to PTH. YAP^Y428F^ mutation also prevented the nuclear translocation of YAP, YAP target genes’ expression, and both OB and Adi differentiation of W-20 BMSCs. In contrast, mimicking Y428 phosphorylation by substitution with aspartic acid (Y428D) stabilized YAP. This unique role of YAP^Y428^ in regulating YAP stability and in the response to PTH was confirmed by the observation that exposing the phosphorylation-deficient YAP^S381A/Y375F^ mutant cells to PTH resulted in the same responses as in wild-type cells. Thus, the effect of PTH on YAP stability, and thereby on BMSC differentiation into OB and Adi, is YAP^Y428^ phosphorylation dependent.

The detailed biochemical analysis of the changes induced by PTH in YAP phosphorylation provides further insight into the mechanisms by which YAP stability is regulated and the role of YAP^Y428^ phosphorylation. PTH decreased markedly YAP^S127^ phosphorylation, an event expected to stabilize YAP (66–68), and decreased the interaction of YAP with LATS1 and with β-TrCP, ultimately preventing efficient phosphorylation of YAP^S127^ and the subsequent ubiquitination and degradation of YAP, respectively (33, 69, 70). Importantly, this series of events is dependent on the presence and phosphorylation of YAP^Y428^, since preventing the steady-state or the PTH-induced phosphorylation of YAP^Y428^ had the opposite effect, favoring the interaction of YAP with LATS1 and β-TrCP, leading to increased YAP^S127^ phosphorylation and YAP ubiquitination. A reasonable hypothesis is, therefore, that the phosphorylation of YAP^Y428^ induces conformational changes that make the LATS1 binding site cryptic and inaccessible for binding. Thus, via the phosphorylation of YAP^Y428^, PTH keeps the Hippo pathway off.

What are the roles of YAP and YAP^Y428^ phosphorylation in the responses of BMSCs to PTH? YAP^Y428^ phosphorylation is shown here to be essential for the W-20 differentiation into both OBs and Adi. This could be attributed to the fact that mutation at YAP^Y428^ impairs YAP nuclear translocation and reduces the expression of YAP target genes that are associated with cell differentiation processes, as well illustrated by our bulk RNA-Seq analysis. Indeed, among the transcriptional targets of YAP, we found that *Ctgf*, *Cyr61*, and *Myc* expression was significantly reduced compared with YAP^Y375F^ and YAP^S381A^ mutant cells. These YAP target genes have been reported to regulate cell differentiation of mesenchymal stromal cells (71). In fact, *Ctgf* and *Cyr61* belong to the cellular communicating network family of secreted extracellular matrix proteins that regulate many tissues, as well as BMSC differentiation (72–74). However, since our results show that YAP^Y428^ mutation impairs both the OB and Adi lineages, the phosphorylation of YAP^Y428^ may be required for BMSC differentiation into both OBs and Adi, i.e., before the branching point of cell fate decision into one or the other lineage.

This would suggest that by stabilizing YAP, PTH favors the progression of BMSCs along their early differentiation but that the PTH-induced impairment of adipogenic differentiation at the point of cell fate decision is mediated by a different mechanism. Of note, we observed the same effects of PTH on YAP stability in 2 osteocyte cell lines, suggesting that YAP regulation in response to PTH also plays a role in more mature cells, although the effects of YAP stabilization in that cell type have not yet been explored. Although it has been reported that YAP/TAZ allow BMSC commitment toward the osteoblastic lineage while inhibiting adipogenesis (47, 75) it should be noted that in these studies both YAP and TAZ were deleted to activate the Hippo pathway. In fact, our results show that YAP and TAZ are not entirely redundant given that by mutating and destabilizing YAP alone we observe significant changes in cell differentiation despite the presence of TAZ. Others have also shown that in contrast with TAZ, YAP inhibits Runx2 activity in the OB-like cells ROS 17/2.8 (63), confirming the difference between YAP and TAZ in OB differentiation and supporting the idea that decreasing total YAP protein levels (hereby favoring degradation) has negative effects on OB differentiation, as also illustrated by mouse knockouts. Conditional deletion of YAP in fully differentiated osteoblasts from *YAP^ﬂ/ﬂ^* *Ocn*-Cre mice resulted in bone loss associated with decreased OB proliferation and differentiation (32). Thus, YAP may promote the differentiation of stromal cells but inhibit the activity of fully differentiated OBs/osteocytes (31, 76).

Finally, our bulk RNA-Seq analysis establishes clearly that YAP^Y428^ mutation, preventing its phosphorylation in response to PTH, has not only striking effects on the expression and stability of YAP but also profound effects on the transcriptome of W-20 BMSC cells in their response to PTH. Multiple genes related to YAP function are significantly altered, and striking changes also occur in gene sets associated with osteogenic and adipogenic differentiation, supporting the cellular assays’ results. Furthermore, the expression of several genes known to be important for stromal cell differentiation among these gene sets is significantly altered by the YAP^Y428^ mutation, both in the absence and in the presence of PTH. Taken together with our cellular and biochemical results, these findings establish firmly the importance of YAP^Y428^ phosphorylation in the response of BMSCs to PTH.

This study has, however, several limitations. The first is the fact that all our experiments have been done in vitro and using cell lines, predominantly the W-20 BMSC line. The fact that we found similar YAP phosphorylation and stability changes in response to PTH in 2 osteocyte cell lines, however, demonstrates that this response to PTH is not limited to one cell line or cell type in bone. The lack of in vivo data remains nevertheless a limitation, and it will be of great interest to introduce the YAP^Y428^ mutation in mice and determine the effects it has on the skeleton and its response to PTH treatment in vivo. The second limitation is that we analyzed and mutated only YAP in these cells, allowing TAZ to potentially compensate for some of the effects of YAP degradation. This design, however, allowed us to determine that YAP has unique functions that are not compensated for by TAZ.

In conclusion, this study demonstrates in 2 cell types (BMSCs and osteocytes) that activation of PTH signaling increases YAP expression and prevents its degradation, thereby negatively affecting Hippo signaling. By stabilizing YAP in target cells, PTH treatment favors its translocation to the nucleus and transcriptional activity, countering Hippo signaling. We show that the stabilization of YAP is due to the activation of a potentially novel PLC/Ca^2+^/Src pathway that leads to the tyrosine phosphorylation of YAP, in particular Y428. Preventing the phosphorylation of this tyrosine residue led to rapid degradation of YAP and blocked the differentiation of BMSCs into both the OB and the Adi lineages. These results suggest that the effects of PTH on its target cells include a repression of the Hippo pathway, generated by the PTH1R by the activation of Src kinase via the PLC/Ca^2+^ pathway, resulting in the tyrosine phosphorylation of YAP, which leads to its stabilization, translocation to the nucleus, and increased transcriptional activity.

## Methods

### Sex as a biological variable.

As the work was performed in cell lines, sex was not considered as a biological variable in our studies.

### Cell lines and reagents.

Experiments were performed using W-20 BMSC line donated by Vicki Rosen (Harvard School of Dental Medicine, Boston, Massachusetts, USA) and maintained and cultured as previously described (77). OmGFP66 cells were donated by Sarah Dallas (University of Missouri, Kansas City, Missouri, USA) and maintained and cultured as previously described (78). OCY454 cells were donated by Paola Divieti (Massachusetts General Hospital, Boston, Massachusetts, USA) and maintained and cultured as previously described (79). Cells were treated where indicated for 2 hours with 50 nM of PTH (Tocris Bioscience, catalog 3011), for 6 hours with 10 mM of H89 (Tocris Bioscience, catalog 2910), for 6 hours with 1 mM of G06983 (Tocris Bioscience, catalog 2285), for 6 hours with 100 nM of dasatinib (Tocris Bioscience, catalog 6793), for 1 hour with 1 mM of U73122 (Tocris Bioscience, catalog 1268), and for 1 hour with 10 mM of BAPTA-AM (S7534 Selleckchem).

### Generation of cell lines, gateway cloning, and tyrosine site identification.

YAP Src tyrosine phosphorylation consensus sequences 375 and 428 were identified using the software GPS 3.0.exe. We used the pENTR221-YAP as a donor vector/entry vector (Addgene, plasmid 79503) and the pLenti PGK Puro DEST (w529-2) as a destination vector (Addgene, plasmid 19068). Stable W-20 YAP(S381A) was generated by using the plasmid pCMV-Flag-YAP-S381A (Addgene, plasmid 33090). We used the pCMV-Flag-YAP-S381A (Addgene, plasmid 33090) as a backbone to generate the stable W-20 YAP(S381A/Y375F) cell line and W-20 YAP (S381A/Y428F by using the Q5 Site-directed Mutagenesis Kit (New England Biolabs E0554S). We used the p2xFlag CMV2-YAP (Addgene, plasmid 19044) as a backbone to generate the W-20 YAP Y375F mutation, W-20 YAPY3428F, and W-20 YAP428D cell lines. For all the above-listed cell lines, positive clones were selected with 100 μg/mL of puromycin and further tested for the presence of the FLAG-tag protein (contained in the N-terminal of all the plasmids used) by Western blot analysis.

### Western blot analysis and cytoplasmic/nuclear extraction.

Protein concentration was determined using Micro BCA Protein Assay Reagent kit (Rockford). Equal amounts of total proteins (5 μg) were resolved by SDS-PAGE under reducing conditions. Cytoplasmic and nuclear fractions were prepared using the Abcam kit (ab113474) according to the manufacturer’s instructions and an equal amount of total proteins (25 μg) were resolved by SDS-PAGE under reducing conditions.

We used ph-YAP (Ser127) (Cell Signaling Technology [CST] 13008), YAP (CST 14074), β-Tubulin (CST 2146), Histone H3 (CST 5192), pan-TEAD (CST 13295), Lamin A/C (CST 2032), FLAG (CST 14793), ph-Src family (Tyr 416) (CST 2101), β-TrCP (CST 4394), ubiquitin (CST 58395), β-actin (SC47778) (Santa Cruz Biotechnology Inc.), and LATS (20276-1-AP) (Proteintech). The polyclonal anti-rabbit antibody against ph-YAPY428 was generated by ProteoGenix. New Zealand white rabbits were immunized against either the unphosphorylated sequence peptide SQQSRFPDYLEA or to the same peptide but where the Y428 was phosphorylated in vitro (SQQSRFPDY*LEA) conjugated to the KLH carrier using a standard protocol (ProteoGenix). Briefly, rabbits received subcutaneous injections of the SQQSRFPDYLEA or the SQQSRFPDY*LEA peptide supplemented with Freund’s adjuvant. After 70 days, the serum was collected and purified. Polyclonal antibodies were collected in PBS supplemented with sodium azide, and concentration was determined with the A280 method. Anti-mouse IgG HRP (CST 7076) and anti-rabbit IgG-HRP (CST 7074) were used as secondary antibodies (Cell Signaling Technologies). Immunoreactivities were assessed using Immobilon Western Chemiluminescent HRP Substrate (WBKLS0100) (MilliporeSigma). The protein bands were analyzed using the ImageJ program (NIH). Protein levels were normalized against housekeeping proteins depending on the assay, and the treated samples were compared against nontreated controls resulting in relative protein levels (fold change against control). Spliced bands are part of the same gel. Data are expressed as fold change, and protein levels were normalized to the respective housekeeping protein in the same sample.

### Immunoprecipitation analysis.

Immunoprecipitation was performed according to the Abcam kit (ab206996) instructions. Briefly, a total of 0.8–1 mg of protein (from the whole cell lysate) or 0.2–0.5 mg of protein (from the cytoplasmic/nuclear extract) were used for each pull-down reaction with the indicated antibodies at 4°C overnight, followed by incubation with protein A agarose beads at 4°C for 2 hours. After washing, bound proteins were eluted and used for Western blot analysis.

### Quantitative-real time PCR.

Total RNA was isolated from cells using the RNeasy Mini Kit (QIAGEN) according to the manufacturer’s protocols, and cDNA was synthesized using iScript cDNA Synthesis Kit (Bio-Rad Laboratories). RT-qPCR was performed using iTaq SYBR Green Supermix (Bio-Rad Laboratories). Relative expression of the genes of interest was done by using the 2^-ΔΔCT^ method (80). Data are expressed as fold change relative to vehicle, and gene expression was normalized to GAPDH housekeeping gene in the same sample.

### Bulk RNA-Seq analysis.

Total RNA was isolated from bone marrow stromal W-20 cells using the RNeasy Mini Kit according to the manufacturer’s protocols. RNA samples with RNA integrity number above 6 were selected for library preparation. Sequencing was performed by Novogene. FASTQ files were mapped using STAR ver. 2.7.10b (81) on the primary assembly mouse genome GRCm39 and basic gene annotation vM31 downloaded from GENCODE (82). The gene expression levels were summarized in a count matrix using RSEM (83). Genes with 10 or more counts were filtered and normalized using DESeq2 (84). PCA was performed using prcomp in stats (85). The distribution of log fold changes in gene expression and adjusted *P* values were drawn on volcano plots, and appropriate threshold values were determined. Significantly differentially expressed genes were summarized in heatmaps referring to interesting Gene Ontology terms in Molecular Signatures Database (MSigDB) (m2.cp.v2023.1.Mm.symbols.gmt). The AUC scores calculated using AUCell were scaled for each term (86). The list of YAP-related genes has been built by referring to the curated gene set in the public database MSigDB (m5.all.v2023.1.Mm.symbols.gmt) (86), the human Hippo YAP gene set https://www.gsea-msigdb.org/gsea/msigdb/human/geneset/WP_HIPPOYAP_SIGNALING.html, and a mouse Hippo signaling gene set https://www.gsea-msigdb.org/gsea/msigdb/mouse/geneset/GOBP_HIPPO_SIGNALING.html, together with some review papers (87, 88).

### OB and Adi differentiation.

Cells were seeded at a density of 2 × 10^4^ and differentiated in osteoblastic medium containing β-glycerophosphate 10 mM, ascorbic acid 50 mg/mL, and BMP2 1,000 ng as previously described (79). At the indicated time, cells were fixed with 4% paraformaldehyde, and Alp staining was performed with 1.5 M Tris-HCl (pH 8.8), NapthThd-AS-MX (Sigma), Fast Blue RR salt (Sigma), and *N,N*-diisopropylethylamine (Sigma). For adipocyte differentiation, cells were seeded at a density of 5 × 10^4^ and cultured in adipocyte differentiation medium (3-isobutyl-l-methylxanthine 500 mm, dexamethasone 1 mm, insulin 10 mg/mL, and rosiglitazone 1 mM, Sigma). After 2 days, cells were cultured in adipocyte differentiation base medium (insulin 10 mg/mL and rosiglitazone 1 mM) for 14 days. Afterward, cells were fixed with 10% formalin, and Oil Red O staining was performed with Oil Red O diluted in water for 30 minutes. Cells were then rinsed with 60% propranolol and finally with water. After staining, the plates were scanned using a flatbed scanner Epson Perfection V600 under identical settings for all samples to ensure consistent image acquisition. The quantification was performed using ImageJ software.

### Statistics.

Data are expressed as the mean ± SEM of at least 3 independent experiments. Statistical analysis was conducted with GraphPad Prism version 10 using unpaired 2-tailed Student’s *t* test or 1-way ANOVA followed by Tukey’s test for multiple comparisons as detailed in each figure legend. A 2-sided *P* value of less than 0.05 was considered the threshold for statistical significance.

### Study approval.

As the work was performed in cell lines, study approval was not necessary.

### Data availability.

Data are available in the Supporting Data Values XLS file or from the corresponding author upon request. Bulk RNA-Seq data have been deposited in the NCBI GEO repository (GSE284675).

## Author contributions

SM, MW, FG, and RB designed research studies; SM, MW, KM, and BRK conducted experiments; SM, MMRDS, and HO analyzed RNA-Seq data; SM, FG, and RB wrote the paper; and SM, CJR, FG, and RB edited the manuscript.

## Supplementary Material

Supplemental data

Unedited blot and gel images

Supporting data values

## Version 1 07/22/2025

In-Press Preview

## Version 2 08/22/2025

Electronic publication
