# Supplementary material for: PTH counteracts Hippo signaling via Src-dependent YAP stabilization to enhance bone marrow stromal cell differentiation
Source: JCI Insight. 2025 Jul 22;10(16):e191245. doi: 10.1172/jci.insight.191245 (PMC12406730; doi:10.1172/jci.insight.191245)
Supplement: Supplemental data [file jciinsight-10-191245-s170.pdf]

Supplementary Figures

Supplementary figure 1

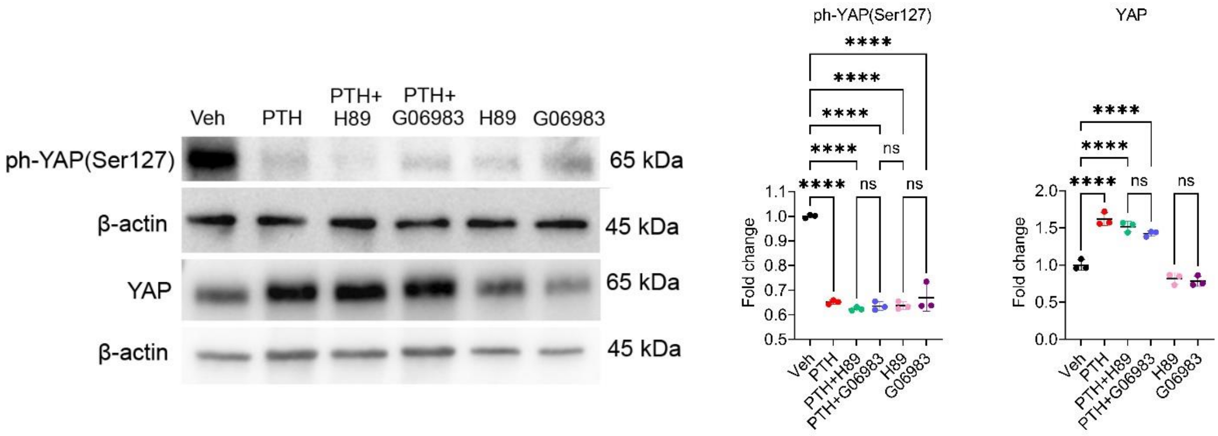

Supplementary Figure 1. PKA and PKC pathways are not involved in PTH-dependent YAP stabilization A. Western analysis representative blots and quantification of ph-YAP(S127) and with or without PTH (50 nM), H89 (10uM), G06983 (1uM), or the combination of the two W-20 cell line. Data are shown as the mean ± SEM of 3 independent experiments. ****p<0.0001 by one-way ANOVA followed by Tukey test for multiple comparisons. The fold change is relative to the Veh.

Supplementary figure 2

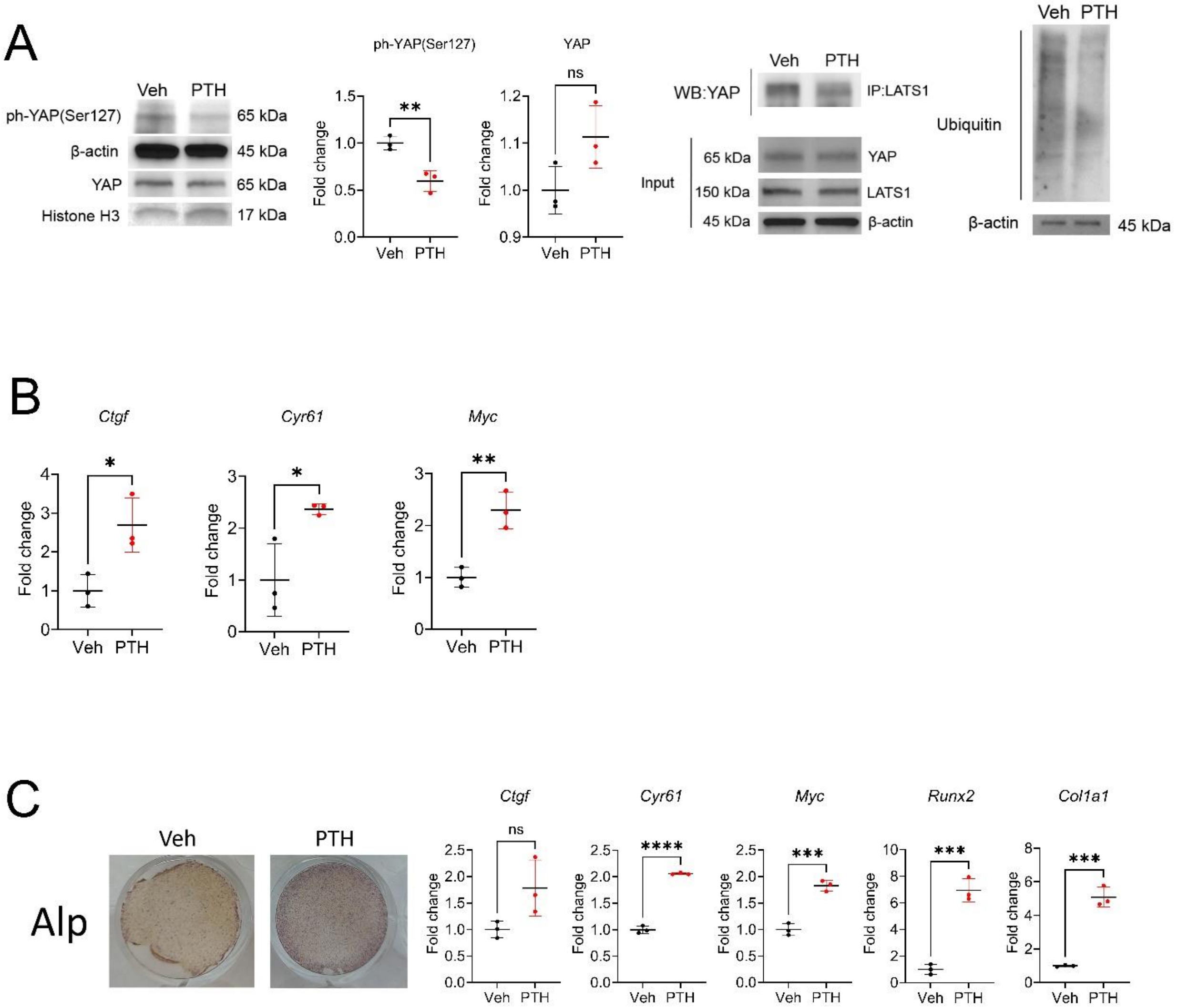

Supplementary Figure 2. PTH promotes YAP stability in W-20 YAP^{S381A} cells

A. Western analysis representative blots and quantification of ph-YAP(S127) and YAP with or without PTH treatment. Representative blots of Co-IP of YAP with LATS1 and ubiquitin levels with or without PTH treatment. **B.** Expression of selected YAP target genes with or without PTH treatment in. **C** Representative image of Alp staining, expression of selected YAP target genes and expression of selected osteoblasts markers after OB differentiation with or without PTH treatment. Data are shown as the mean ± SEM of 3 independent experiments. *p<0.05, **p<0.005, ***p<0.0005, ****p<0.0001 by unpaired Student's t-test. The fold change is relative to the Veh.

Panel B mean Ct values: Gene *Ctgf* Veh 25.65; PTH 23.45. Gene *Cyr6*1 Veh 21.87; PTH 20.11. Gene *Myc* Veh 27.70; PTH 27.85. Panel C mean Ct values: Gene *Ctgf* Veh 25.44; PTH 24.40. Gene *Cyr6*1 Veh 22.22; PTH 21.58. Gene *Myc* Veh 24.39; PTH 25.27. Gene *Runx2* Veh 26.97; PTH 24.34. Gene *Col1a1* Veh 21.18; PTH 19.59.

Supplementary figure 3

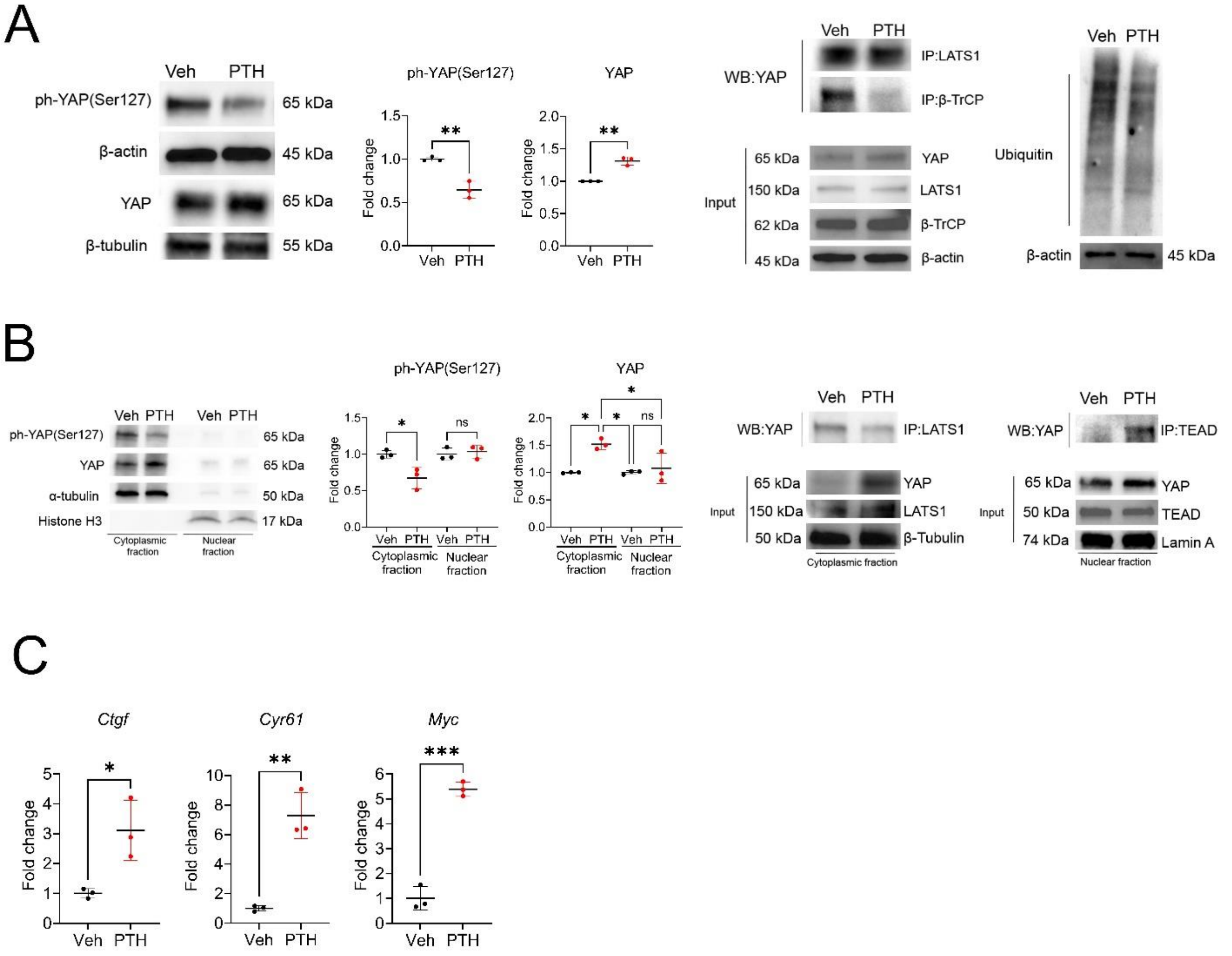

Supplementary Figure 3. PTH promotes YAP stability in $YAP^{S381A/Y375F}$ cells

A. Western analysis representative blots and quantification of ph-YAP(S127) and YAP with or without PTH treatment; representative blots of Co-IP of YAP with LATS1 and β-TrCPand ubiquitin levels with or without PTH treatment. **B**. Western analysis representative blots and quantification of ph-YAP(S127) and YAP protein levels in the cytoplasmic and nuclear fraction with or without PTH treatment and representative blots of Co-IP of YAP with LATS1 in the cytoplasmic fraction and TEAD in the nuclear fraction. **C**. Expression of selected YAP target genes with or without PTH treatment after OB differentiation. Data are shown as the mean ± SEM of 3 independent experiments. *p<0.05, **p<0.005, ***p<0.0005, by unpaired Student's t-test. The fold change is relative to the Veh.

Panel C mean Ct values: Gene *Ctgf* Veh 27.16; PTH 25.22. Gene *Cyr6*1 Veh 23.93; PTH 21.71. Gene *Myc* Veh 22.10; PTH 22.06.

Supplementary figure 4

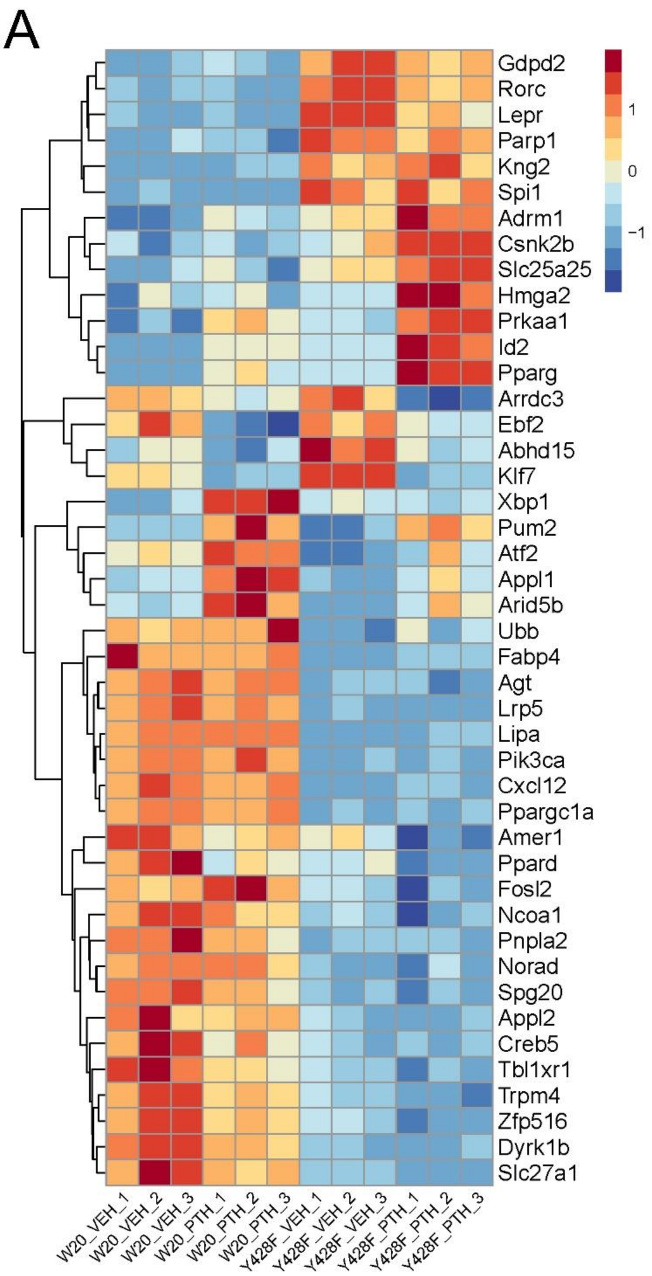

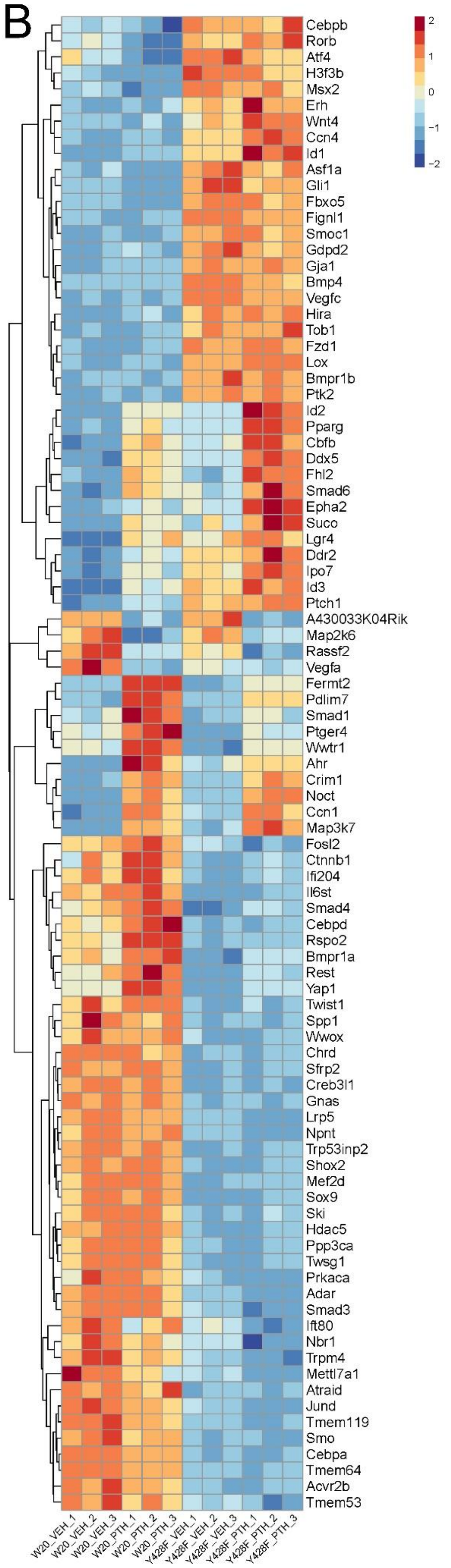

Supplementary Figure 4. PTH differently regulates adipocytes and osteoblast-related genes in W-20 and YAP^{Y428F} cells

A. Heatmap of significantly differentially regulated adipocyte-related genes by PTH in W-20 and in YAP^{Y428F} cells. **B.** Heatmap of significantly differentially regulated osteoblast-related genes by PTH in W0 and in Yap^{Y428F} cells.

Supplementary table 1

Cell Proliferation (O.D. ± SEM)

Time	Ctr	YAP WT	YAP^{S381A}	YAP$^{S381A/Y375F}$	YAP$^{S381A/Y428F}$
**0h**	0,3452 ± 0,0380	0,2754 ± 0,0411	0,3389 ± 0,0513	0,2476 ± 0,0359	0,2967 ± 0,0120
**24h**	0,4187 ± 0,0202	0,3010 ± 0,0505	0,3792 ± 0,0691	0,2802 ± 0,0729	0,4050 ± 0,0338
**48h**	0,8033 ± 0,0999	0,7504 ± 0,1752	1,0452 ± 0,2792	0,6207 ± 0,2317	1,0662 ± 0,0286
**72h**	0,9096 ± 0,0745	0,9142 ± 0,2085	1,1881 ± 0,2808	0,6837 ± 0,2613	1,1118 ± 0,0124
**96h**	1,2918 ± 0,0992	1,6773 ± 0,1190	1,7981 ± 0,1802	1,3606 ± 0,2613	1,7246 ± 0,0124

Multiple comparisons test	Interaction	Adjusted P value
**Ctr vs. YAP WT**	ns	>0,9999
**Ctr vs. YAP^{S381A}**	ns	0,9752
**Ctr vs. YAP$^{S381A/Y375F}$**	ns	0,9967
**Ctr vs. YAP$^{S381A/Y428F}$**	ns	0,9863
**YAP WT vs. YAP^{S381A}**	ns	0,9865
**YAP WT vs. YAP$^{S381A/Y375F}$**	ns	0,9919
**YAP WT vs. YAP$^{S381A/Y428F}$**	ns	0,9935
**YAP^{S381A} vs. YAP$^{S381A/Y375F}$**	ns	0,8801
**YAP^{S381A} vs. YAP$^{S381A/Y428F}$**	ns	>0,9999
**YAP$^{S381A/Y375F}$ vs. YAP$^{S381A/Y428F}$**	ns	0,9124

Supplementary Table 1. YAP mutations do not affect cell proliferation.

CCK-8MTT cell proliferation assay of in W-20 control, YAP WT, YAP^{S381A}, YAP$^{S381A/375F}$ and YAP$^{381A/Y428F}$ cells after indicated time points. ± represent standard error.
