# Supplementary material for: PTH counteracts Hippo signaling via Src-dependent YAP stabilization to enhance bone marrow stromal cell differentiation
Source: JCI Insight. 2025 Jul 22;10(16):e191245. doi: 10.1172/jci.insight.191245 (PMC12406730; doi:10.1172/jci.insight.191245)

Figure 2

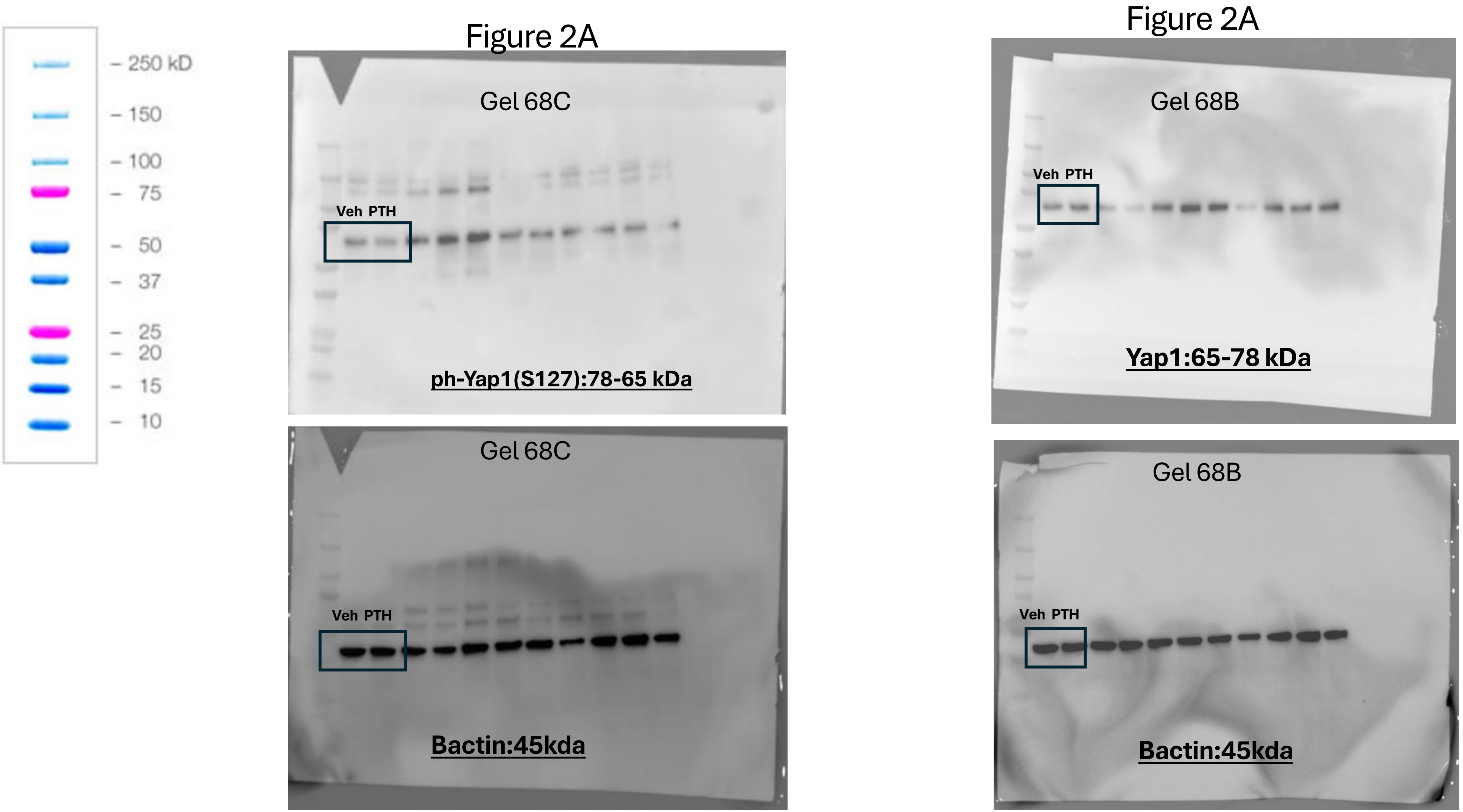

250 kD
150
100
75
50
37
25
20
15
10
Figure 2A
Gel 68C
Veh PTH
ph-Yap1(S127):78-65 kDa
Gel 68C
Veh PTH
Bactin:45kda
Figure 2A
Gel 68B
Veh PTH
Yap1:65-78 kDa
Gel 68B
Veh PTH
Bactin:45kda

Figure 2

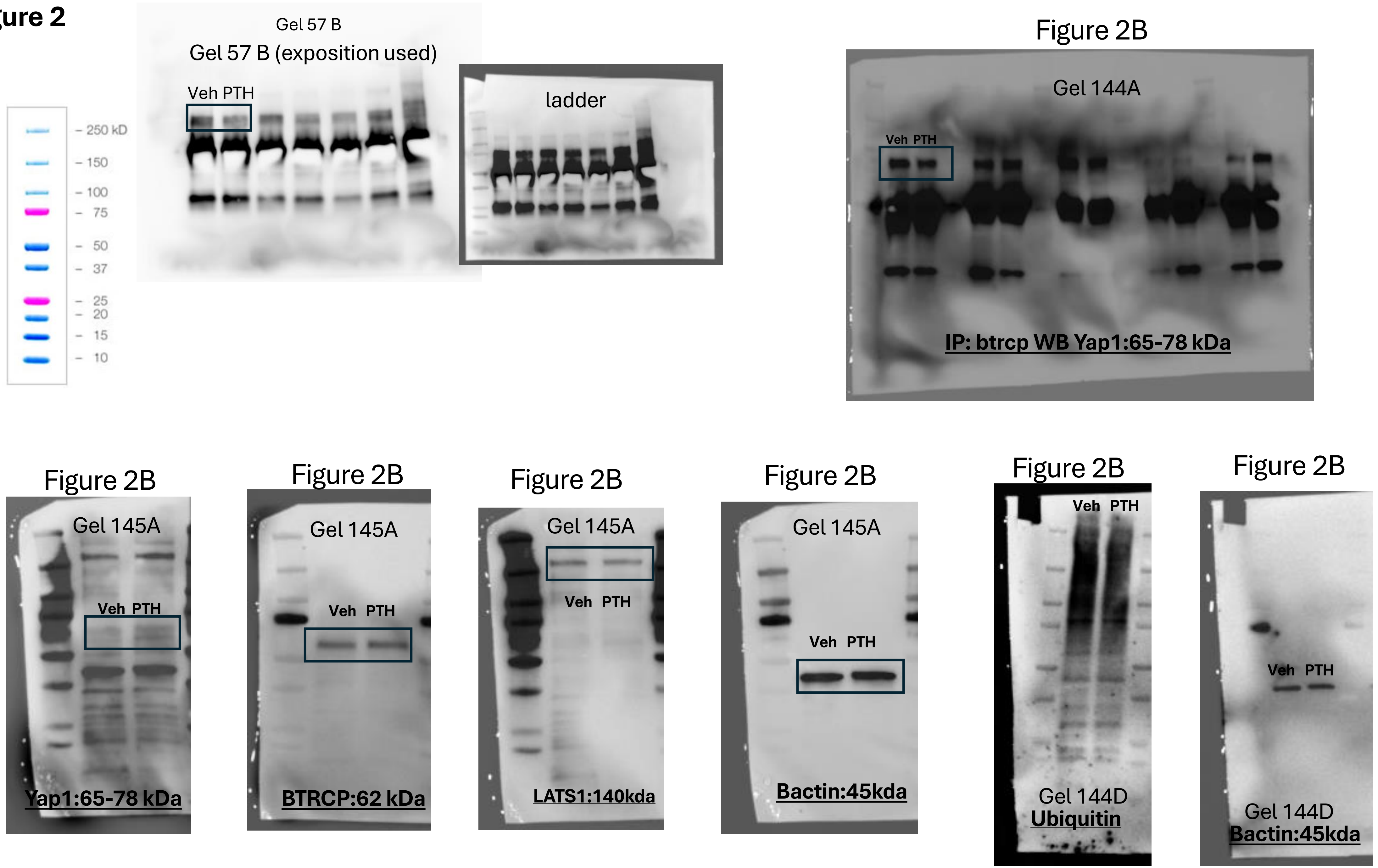
Gel 57 B
Gel 57 B (exposition used)
Veh PTH
250 kD
150
100
75
50
37
25
20
15
10
ladder
Figure 2B
Gel 144A
Veh PTH
IP: btrcp WB Yap1:65-78 kDa
Figure 2B
Gel 145A
Veh PTH
Yap1:65-78 kDa
Figure 2B
Gel 145A
Veh PTH
BTRCP:62 kDa
Figure 2B
Gel 145A
Veh PTH
LATS1:140kda
Figure 2B
Gel 145A
Veh PTH
Bactin:45kda
Figure 2B
Veh PTH
Gel 144D
Ubiquitin
Figure 2B
Veh PTH
Gel 144D
Bactin:45kda

Figure 2

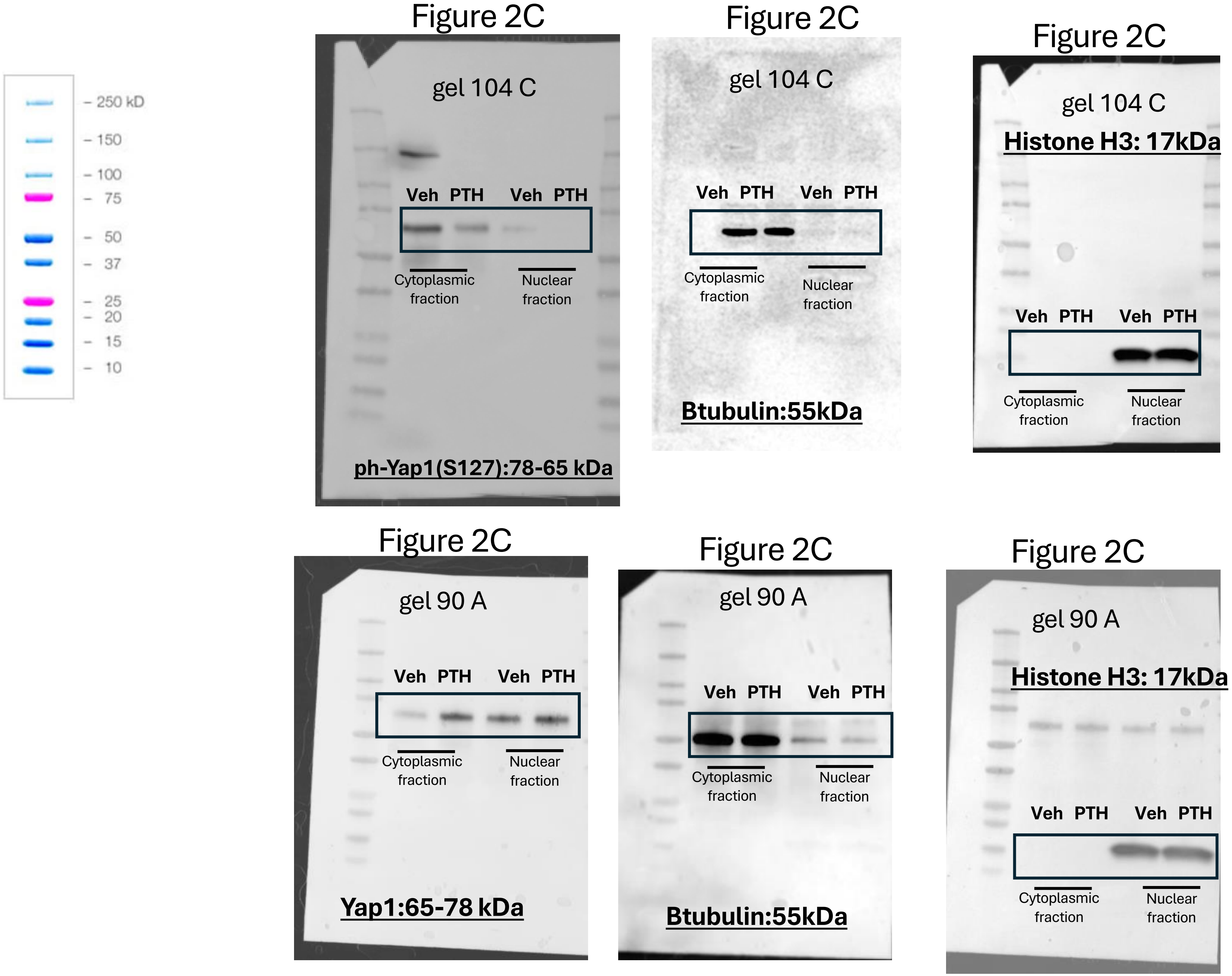

250 kD
150
100
75
50
37
25
20
15
10
Figure 2C
gel 104 C
Veh PTH Veh PTH
Cytoplasmic fraction
Nuclear fraction
ph-Yap1(S127):78-65 kDa
Figure 2C
gel 104 C
Veh PTH Veh PTH
Cytoplasmic fraction
Nuclear fraction
Btubulin:55kDa
Figure 2C
gel 104 C
Histone H3: 17kDa
Veh PTH Veh PTH
Cytoplasmic fraction
Nuclear fraction
Figure 2C
gel 90 A
Veh PTH Veh PTH
Cytoplasmic fraction
Nuclear fraction
Yap1:65-78 kDa
Figure 2C
gel 90 A
Veh PTH Veh PTH
Cytoplasmic fraction
Nuclear fraction
Btubulin:55kDa
Figure 2C
gel 90 A
Histone H3: 17kDa
Veh PTH Veh PTH
Cytoplasmic fraction
Nuclear fraction

Figure 2

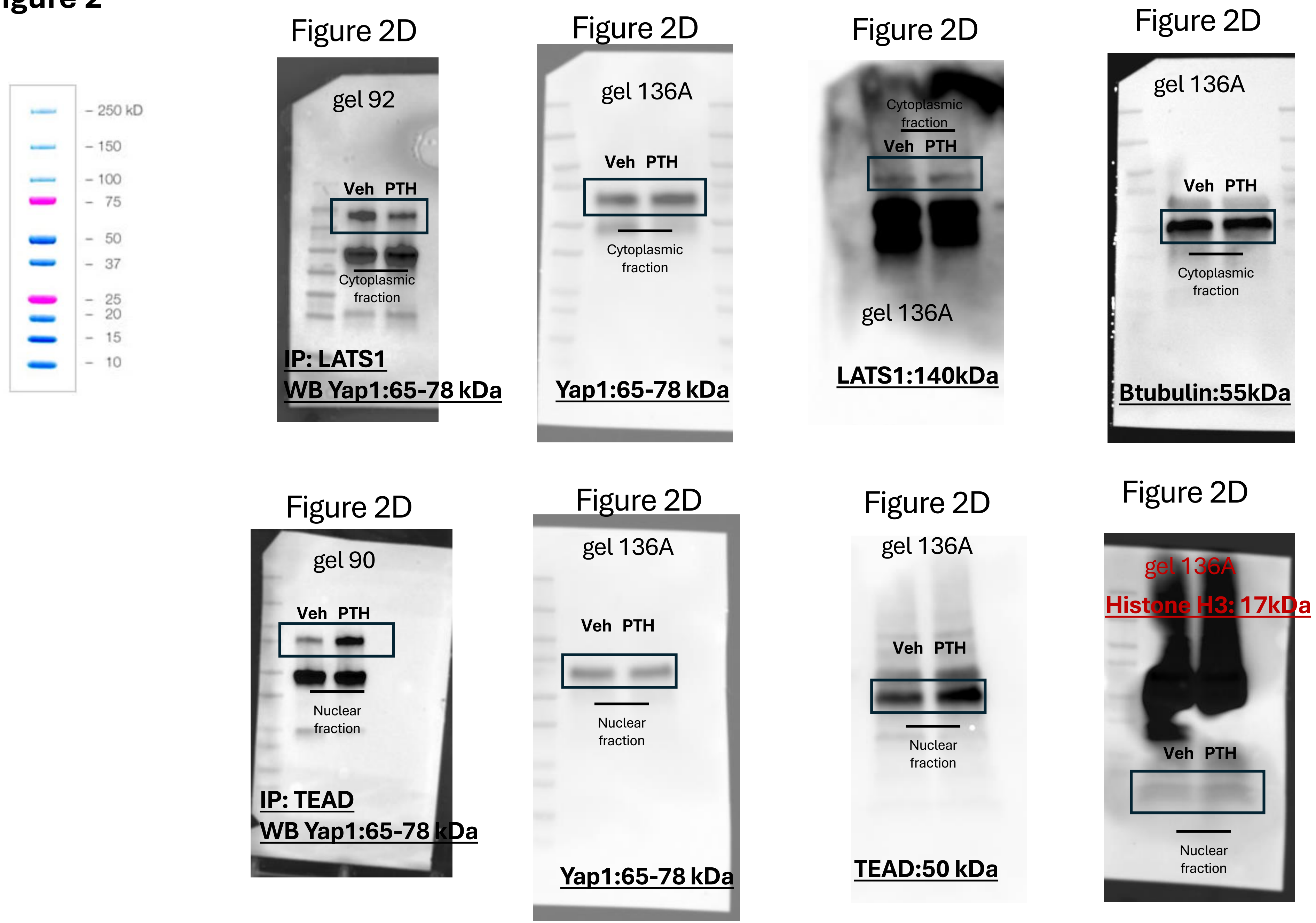

250 kD
150
100
75
50
37
25
20
15
10
Figure 2D
gel 92
Veh PTH
Cytoplasmic fraction
IP: LATS1
WB Yap1:65-78 kDa
Figure 2D
gel 136A
Veh PTH
Cytoplasmic fraction
Yap1:65-78 kDa
Figure 2D
Cytoplasmic fraction
Veh PTH
gel 136A
LATS1:140kDa
Figure 2D
gel 136A
Veh PTH
Cytoplasmic fraction
Btubulin:55kDa
Figure 2D
gel 90
Veh PTH
Nuclear fraction
IP: TEAD
WB Yap1:65-78 kDa
Figure 2D
gel 136A
Veh PTH
Nuclear fraction
Yap1:65-78 kDa
Figure 2D
gel 136A
Veh PTH
Nuclear fraction
TEAD:50 kDa
Figure 2D
gel 136A
Histone H3: 17kDa
Veh PTH
Nuclear fraction

Figure 2

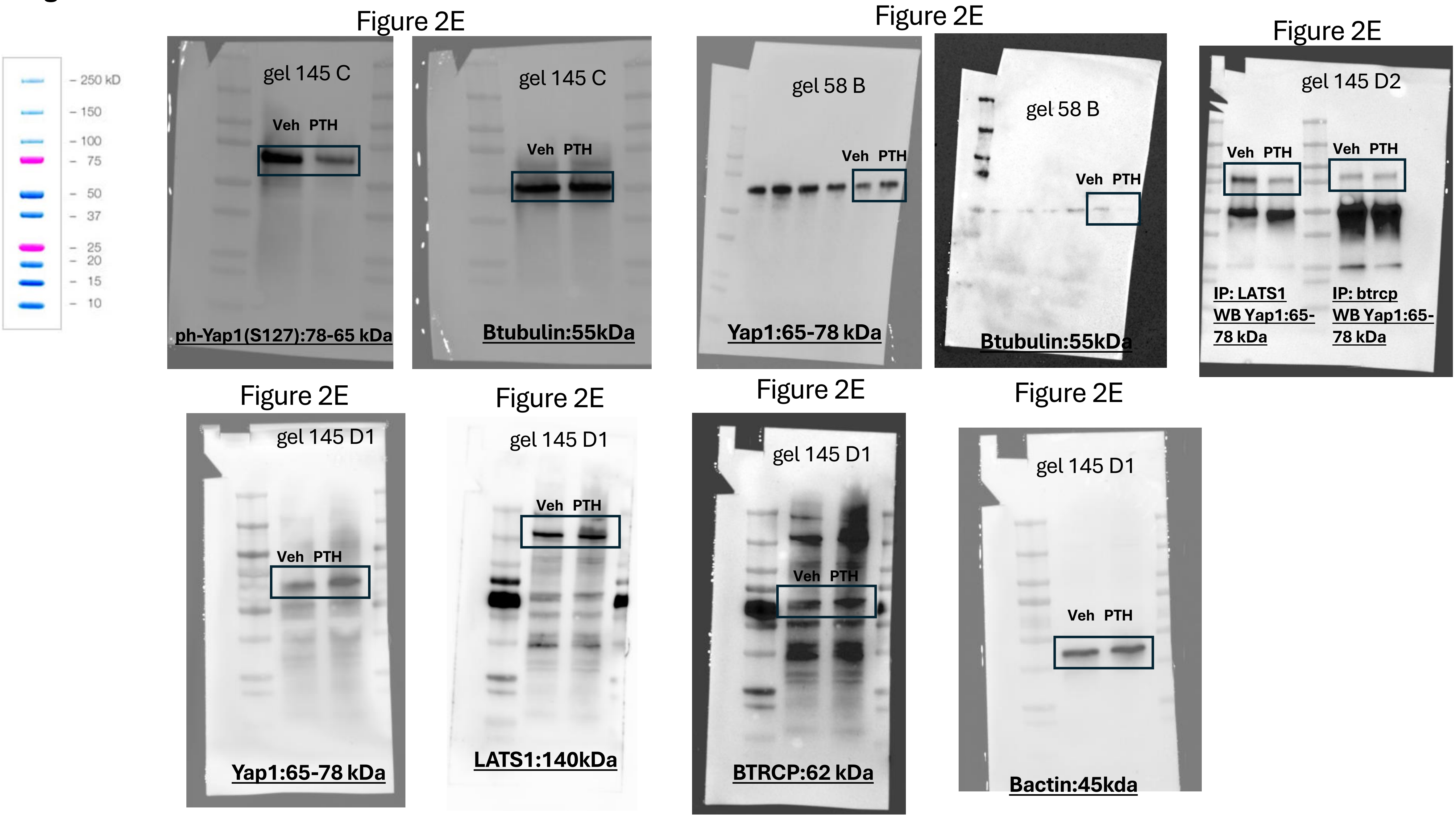

250 kD
150
100
75
50
37
25
20
15
10
Figure 2E
gel 145 C
Veh PTH
ph-Yap1(S127):78-65 kDa
Figure 2E
gel 145 C
Veh PTH
Btubulin:55kDa
Figure 2E
gel 58 B
Veh PTH
Yap1:65-78 kDa
gel 58 B
Veh PTH
Btubulin:55kDa
Figure 2E
gel 145 D2
Veh PTH
Veh PTH
IP: LATS1
WB Yap1:65-78 kDa
IP: btrcp
WB Yap1:65-78 kDa
Figure 2E
gel 145 D1
Veh PTH
Yap1:65-78 kDa
Figure 2E
gel 145 D1
Veh PTH
LATS1:140kDa
Figure 2E
gel 145 D1
Veh PTH
BTRCP:62 kDa
Figure 2E
gel 145 D1
Veh PTH
Bactin:45kda

Figure 2

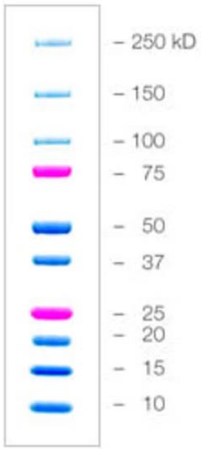

Figure 2G

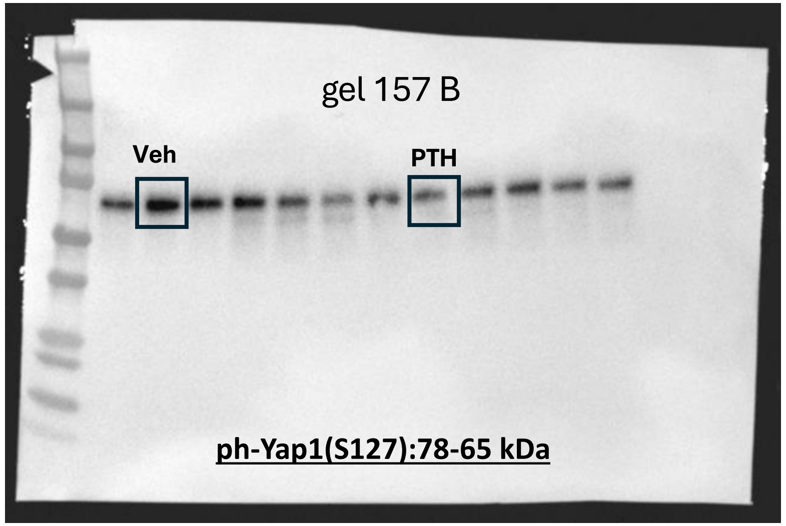

Figure 2G

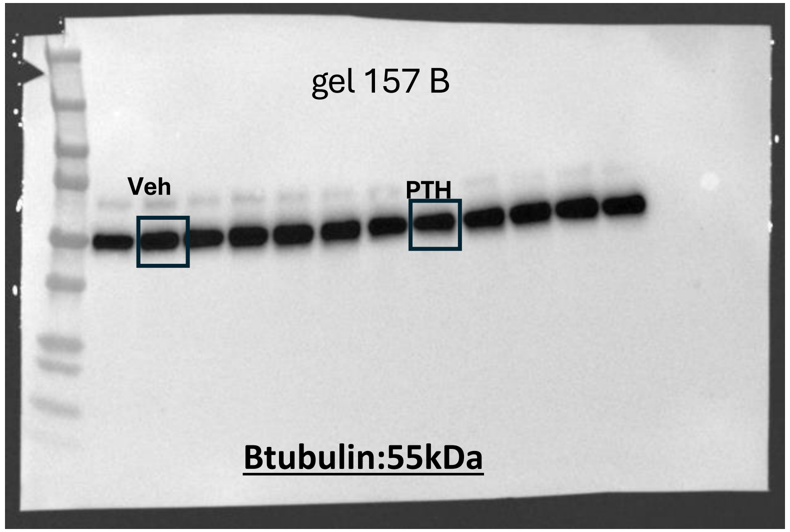

Figure 2G

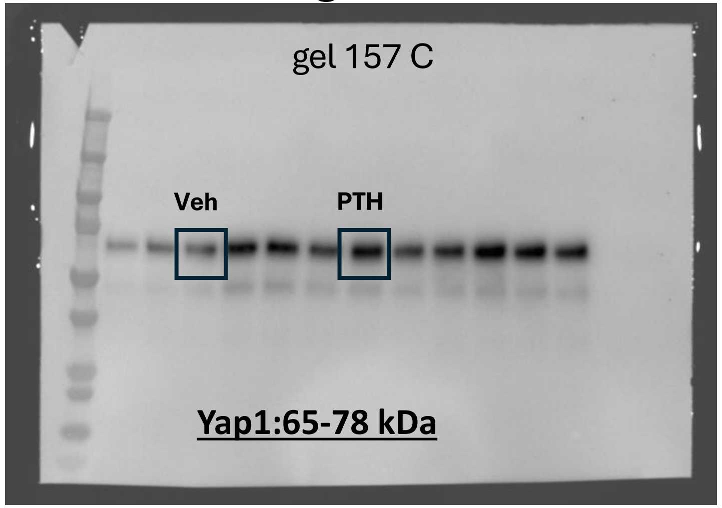

Figure 2G

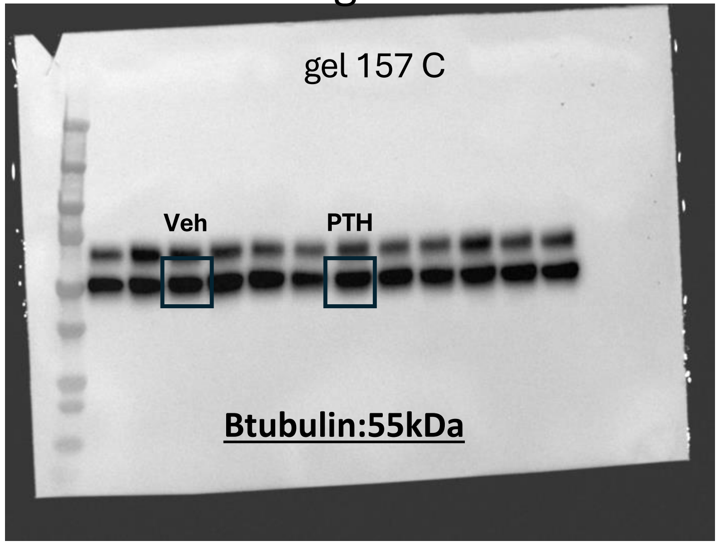

Figure 3

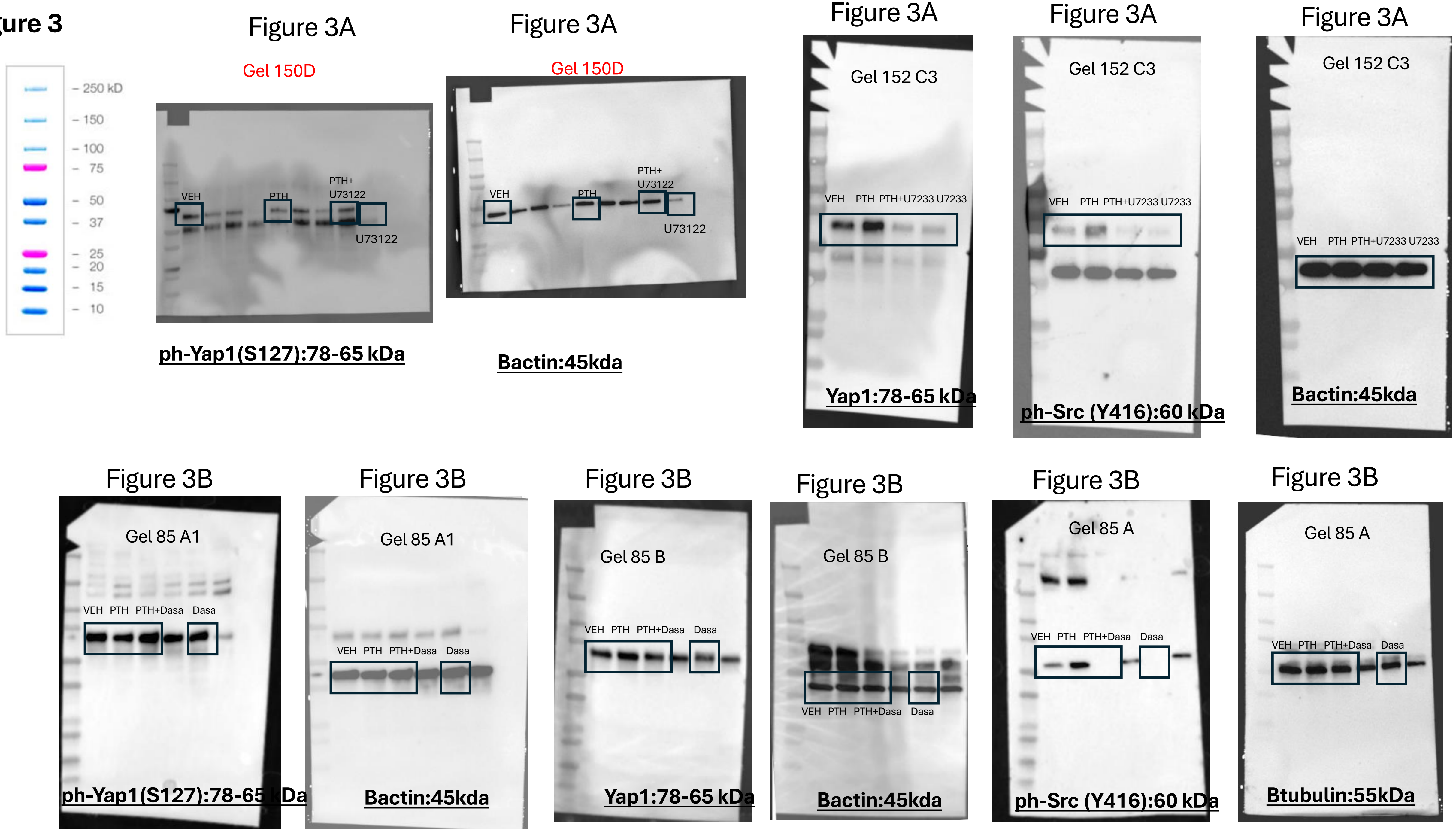
Figure 3A
Figure 3A
Figure 3A
Figure 3A
Figure 3A
250 kD
150
100
75
50
37
25
20
15
10
Gel 150D
VEH
PTH
PTH+
U73122
U73122
ph-Yap1(S127):78-65 kDa
Gel 150D
VEH
PTH
PTH+
U73122
U73122
Bactin:45kda
Gel 152 C3
VEH PTH PTH+U7233 U7233
Yap1:78-65 kDa
Gel 152 C3
VEH PTH PTH+U7233 U7233
ph-Src (Y416):60 kDa
Gel 152 C3
VEH PTH PTH+U7233 U7233
Bactin:45kda
Figure 3B
Figure 3B
Figure 3B
Figure 3B
Figure 3B
Figure 3B
Gel 85 A1
VEH PTH PTH+Dasa Dasa
ph-Yap1(S127):78-65 kDa
Gel 85 A1
VEH PTH PTH+Dasa Dasa
Bactin:45kda
Gel 85 B
VEH PTH PTH+Dasa Dasa
Yap1:78-65 kDa
Gel 85 B
VEH PTH PTH+Dasa Dasa
Bactin:45kda
Gel 85 A
VEH PTH PTH+Dasa Dasa
ph-Src (Y416):60 kDa
Gel 85 A
VEH PTH PTH+Dasa Dasa
Btubulin:55kDa

Figure 3

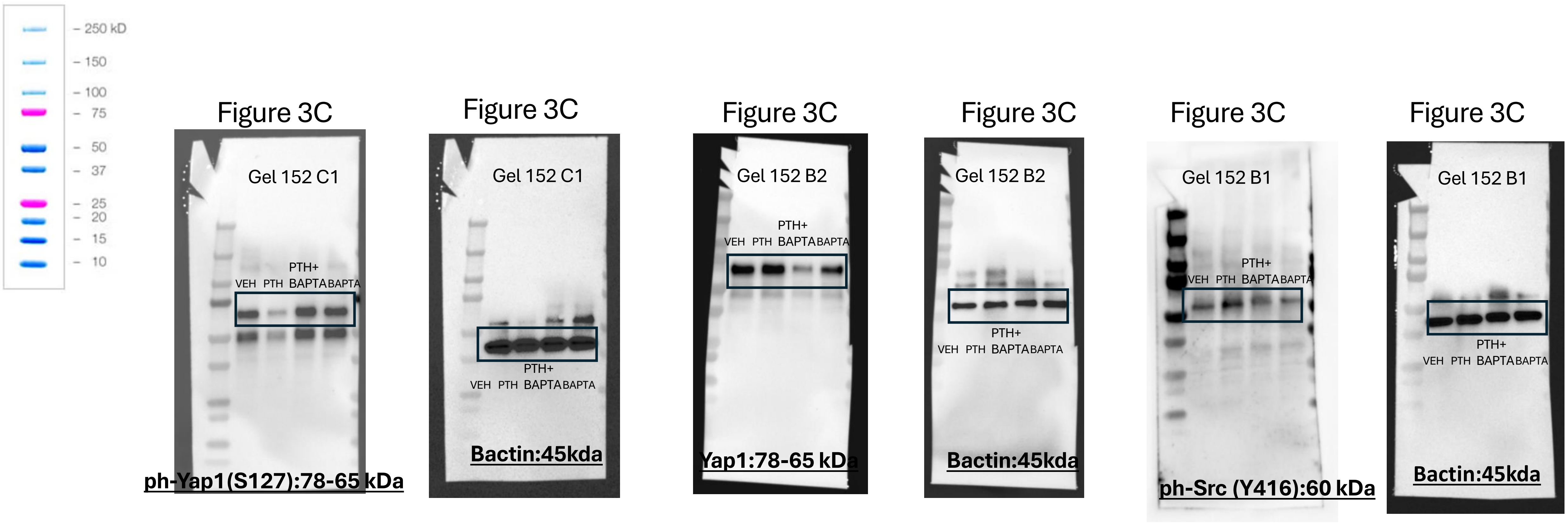

250 kD
150
100
75
50
37
25
20
15
10
Figure 3C
Gel 152 C1
VEH PTH PTH+ BAPTA BAPTA
ph-Yap1(S127):78-65 kDa
Figure 3C
Gel 152 C1
VEH PTH PTH+ BAPTA BAPTA
Bactin:45kda
Figure 3C
Gel 152 B2
VEH PTH PTH+ BAPTA BAPTA
Yap1:78-65 kDa
Figure 3C
Gel 152 B2
VEH PTH PTH+ BAPTA BAPTA
Bactin:45kda
Figure 3C
Gel 152 B1
VEH PTH PTH+ BAPTA BAPTA
ph-Src (Y416):60 kDa
Figure 3C
Gel 152 B1
VEH PTH PTH+ BAPTA BAPTA
Bactin:45kda

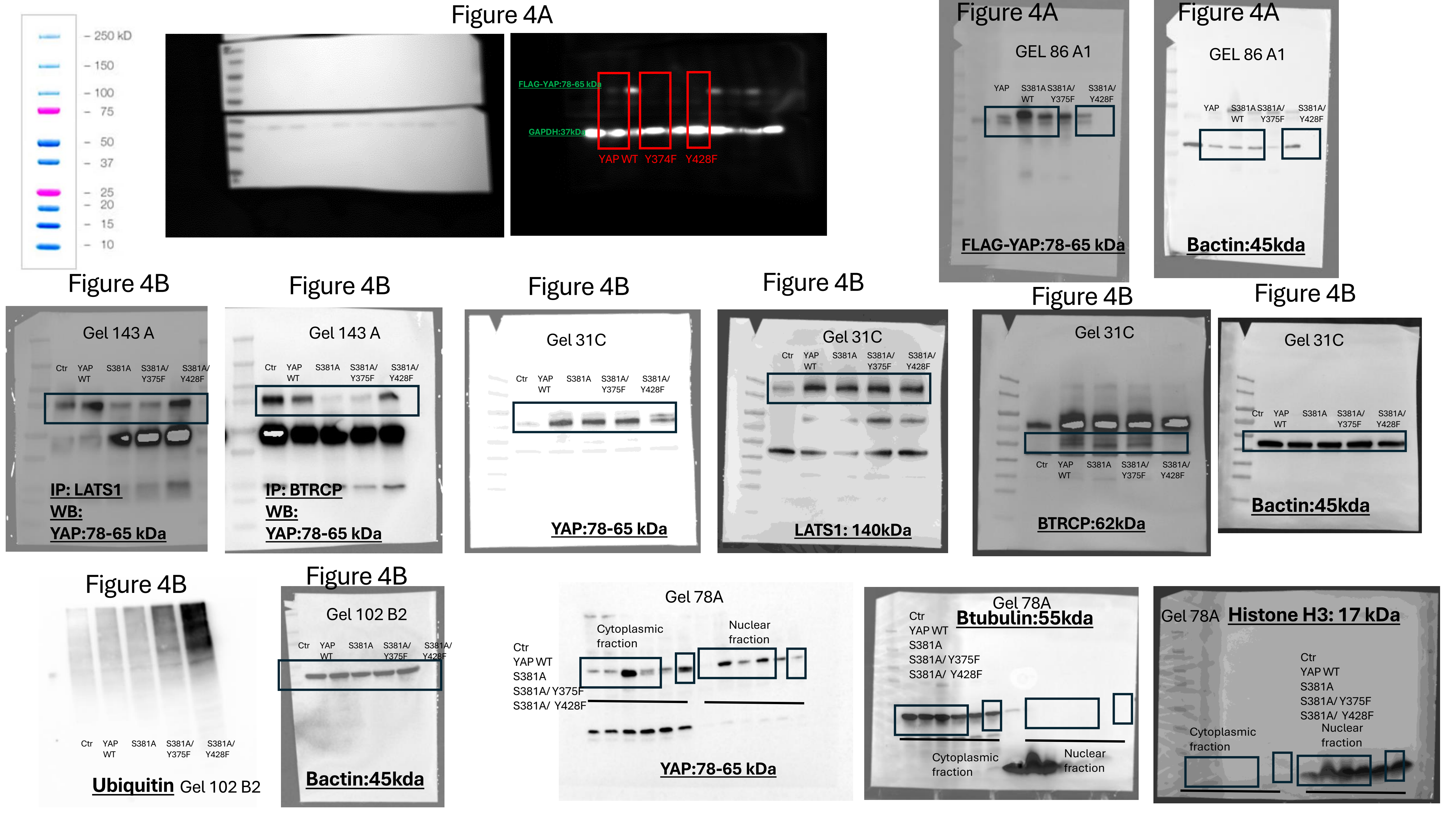
Figure 4A
250 kD
150
100
75
50
37
25
20
15
10
FLAG-YAP:78-65 kDa
GAPDH:37kDa
YAP WT
Y374F
Y428F
Figure 4A
GEL 86 A1
YAP
S381A WT
S381A/ Y375F
S381A/ Y428F
FLAG-YAP:78-65 kDa
Figure 4A
GEL 86 A1
YAP
S381A WT
S381A/ Y375F
S381A/ Y428F
Bactin:45kda
Figure 4B
Gel 143 A
Ctr
YAP WT
S381A
S381A/ Y375F
S381A/ Y428F
IP: LATS1
WB:
YAP:78-65 kDa
Figure 4B
Gel 143 A
Ctr
YAP WT
S381A
S381A/ Y375F
S381A/ Y428F
IP: BTRCP
WB:
YAP:78-65 kDa
Figure 4B
Gel 31C
Ctr
YAP WT
S381A
S381A/ Y375F
S381A/ Y428F
YAP:78-65 kDa
Figure 4B
Gel 31C
Ctr
YAP WT
S381A
S381A/ Y375F
S381A/ Y428F
LATS1: 140kDa
Figure 4B
Gel 31C
Ctr
YAP WT
S381A
S381A/ Y375F
S381A/ Y428F
BTRCP:62kDa
Figure 4B
Gel 31C
Ctr
YAP WT
S381A
S381A/ Y375F
S381A/ Y428F
Bactin:45kda
Figure 4B
Ctr
YAP WT
S381A
S381A/ Y375F
S381A/ Y428F
Ubiquitin
Gel 102 B2
Figure 4B
Gel 102 B2
Ctr
YAP WT
S381A
S381A/ Y375F
S381A/ Y428F
Bactin:45kda
Gel 78A
Ctr
YAP WT
S381A
S381A/ Y375F
S381A/ Y428F
Cytoplasmic fraction
Nuclear fraction
YAP:78-65 kDa
Gel 78A
Btubulin:55kda
Ctr
YAP WT
S381A
S381A/ Y375F
S381A/ Y428F
Cytoplasmic fraction
Nuclear fraction
Gel 78A
Histone H3: 17 kDa
Ctr
YAP WT
S381A
S381A/ Y375F
S381A/ Y428F
Cytoplasmic fraction
Nuclear fraction

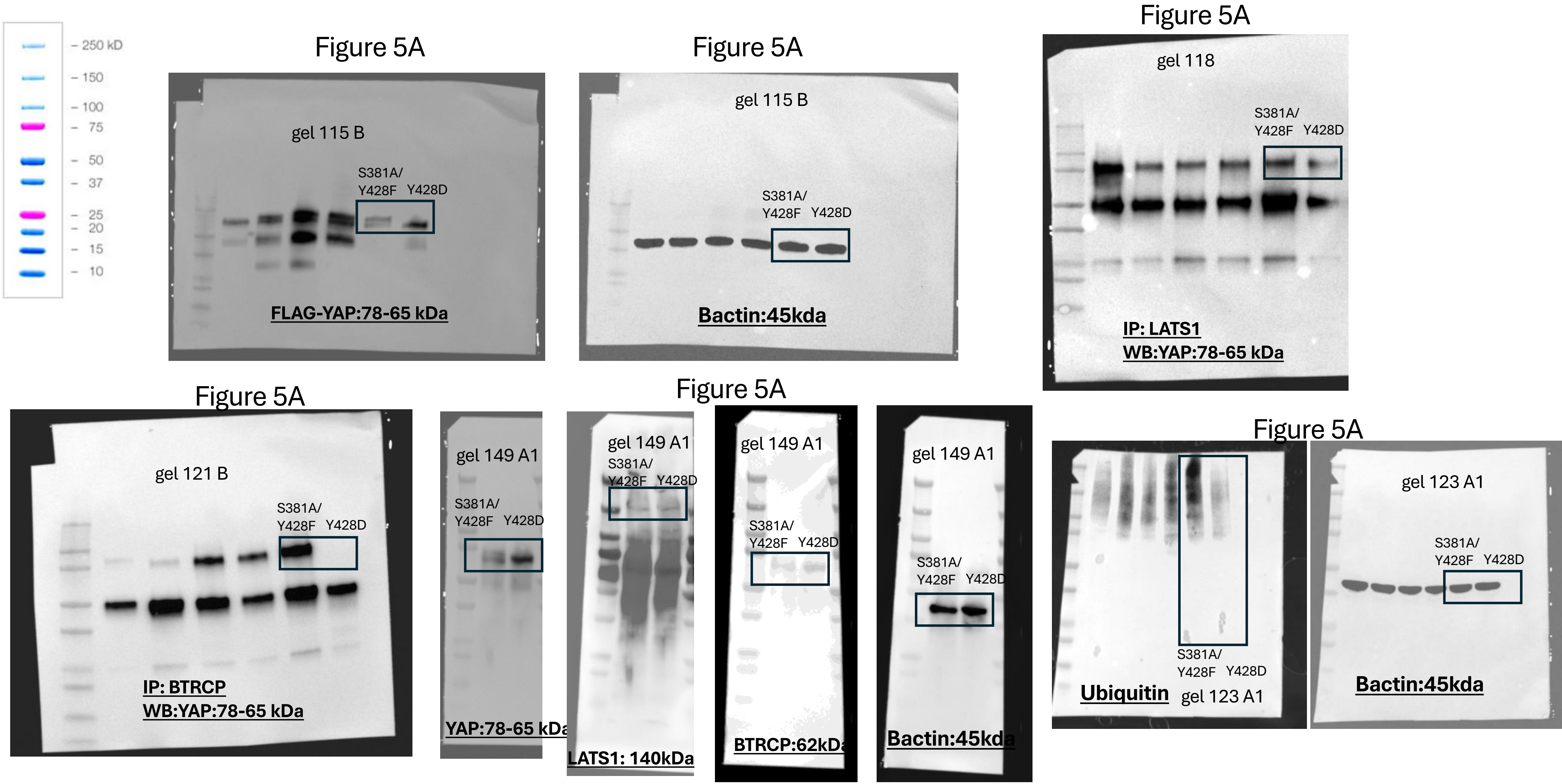

- 250 kD
- 150
- 100
- 75
- 50
- 37
- 25
- 20
- 15
- 10
Figure 5A
gel 115 B
S381A/
Y428F Y428D
FLAG-YAP:78-65 kDa
Figure 5A
gel 115 B
S381A/
Y428F Y428D
Bactin:45kda
Figure 5A
gel 118
S381A/
Y428F Y428D
IP: LATS1
WB:YAP:78-65 kDa
Figure 5A
gel 121 B
S381A/
Y428F Y428D
IP: BTRCP
WB:YAP:78-65 kDa
Figure 5A
gel 149 A1
S381A/
Y428F Y428D
YAP:78-65 kDa
gel 149 A1
S381A/
Y428F Y428D
LATS1: 140kDa
gel 149 A1
S381A/
Y428F Y428D
BTRCP:62kDa
gel 149 A1
S381A/
Y428F Y428D
Bactin:45kda
Figure 5A
S381A/
Y428F Y428D
Ubiquitin
gel 123 A1
gel 123 A1
S381A/
Y428F Y428D
Bactin:45kda

Figure 5B

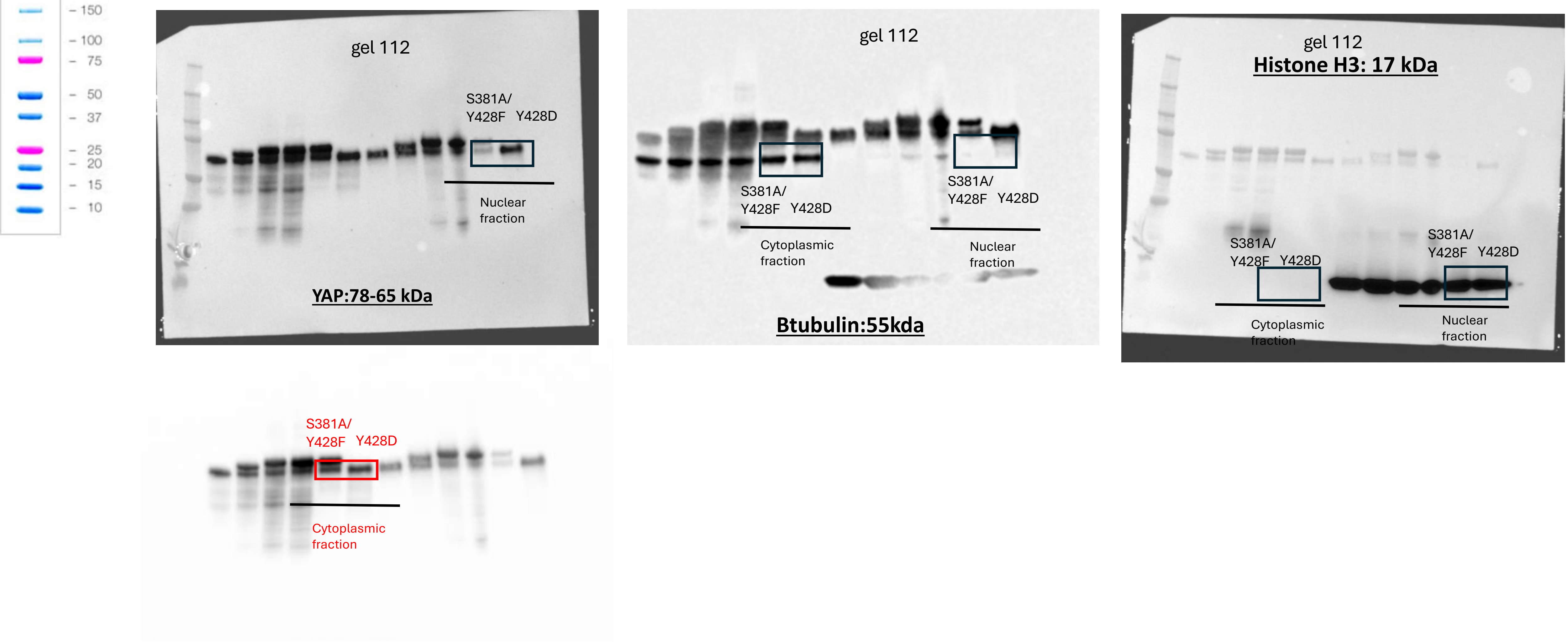

250 kD
150
100
75
50
37
25
20
15
10
gel 112
S381A/
Y428F
Y428D
Nuclear
fraction
YAP:78-65 kDa
gel 112
S381A/
Y428F
Y428D
Cytoplasmic
fraction
S381A/
Y428F
Y428D
Nuclear
fraction
Btubulin:55kda
gel 112
Histone H3: 17 kDa
S381A/
Y428F
Y428D
Cytoplasmic
fraction
S381A/
Y428F
Y428D
Nuclear
fraction
S381A/
Y428F
Y428D
Cytoplasmic
fraction

Figure 6

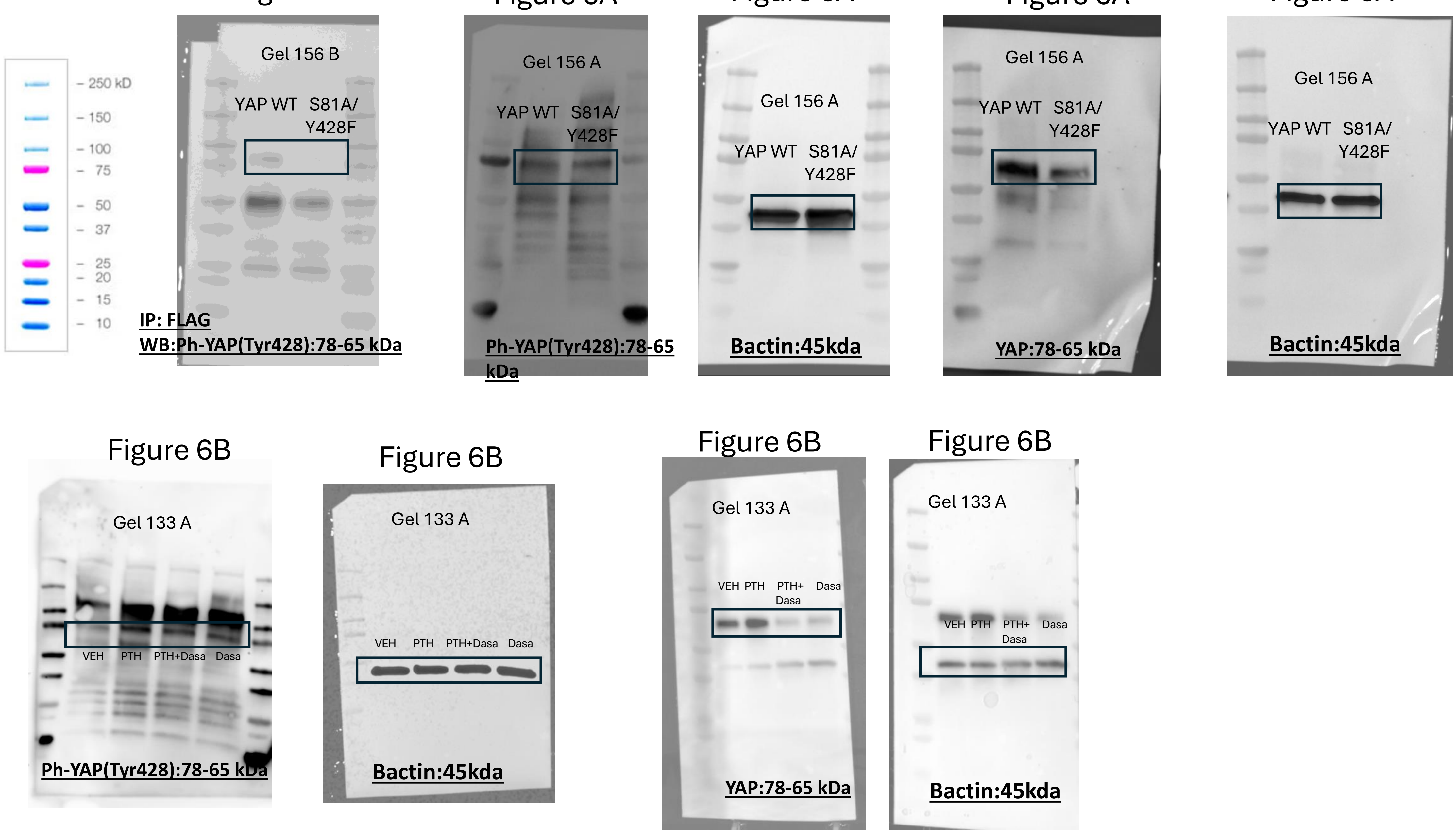

Figure 6A
250 kD
150
100
75
50
37
25
20
15
10
Gel 156 B
YAP WT
S81A/ Y428F
IP: FLAG
WB:Ph-YAP(Tyr428):78-65 kDa
Figure 6A
Gel 156 A
YAP WT
S81A/ Y428F
Ph-YAP(Tyr428):78-65 kDa
Figure 6A
Gel 156 A
YAP WT
S81A/ Y428F
Bactin:45kda
Figure 6A
Gel 156 A
YAP WT
S81A/ Y428F
YAP:78-65 kDa
Figure 6A
Gel 156 A
YAP WT
S81A/ Y428F
Bactin:45kda
Figure 6B
Gel 133 A
VEH
PTH
PTH+Dasa
Dasa
Ph-YAP(Tyr428):78-65 kDa
Figure 6B
Gel 133 A
VEH
PTH
PTH+Dasa
Dasa
Bactin:45kda
Figure 6B
Gel 133 A
VEH
PTH
PTH+ Dasa
Dasa
YAP:78-65 kDa
Figure 6B
Gel 133 A
VEH
PTH
PTH+ Dasa
Dasa
Bactin:45kda

Figure 6

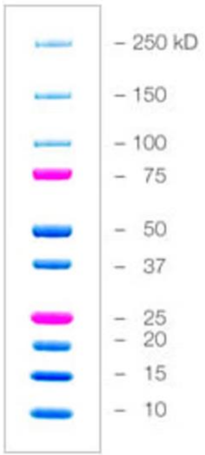

Figure 6C

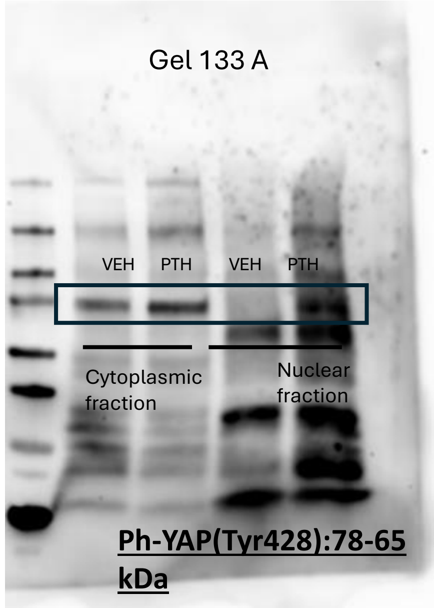

Figure 6C

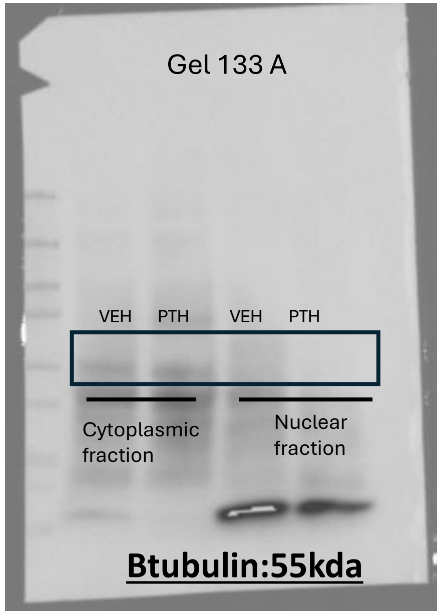

Figure 6C

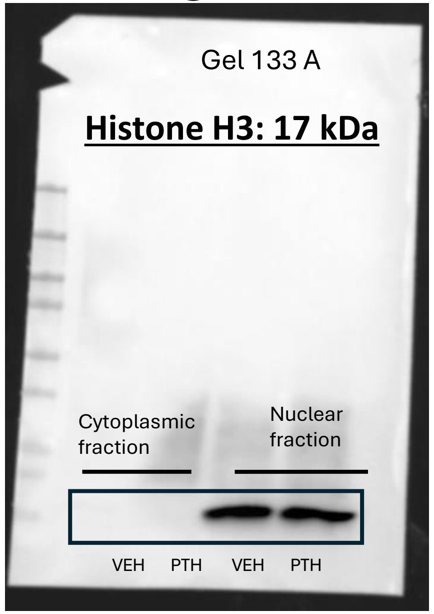

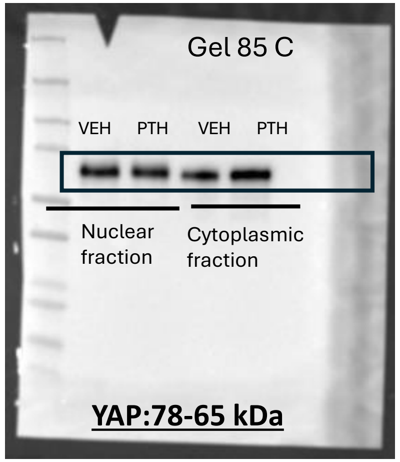

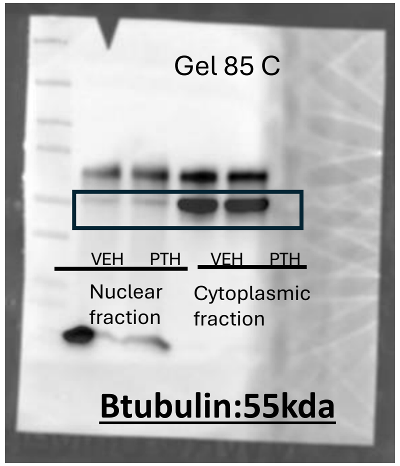

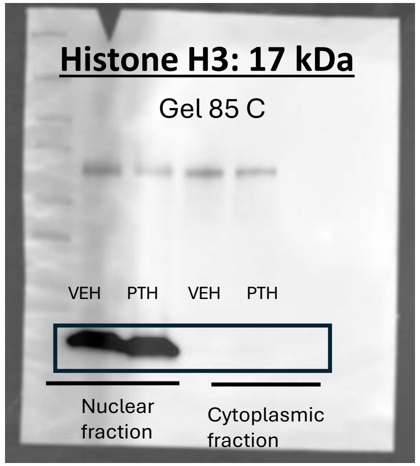

Figure 7

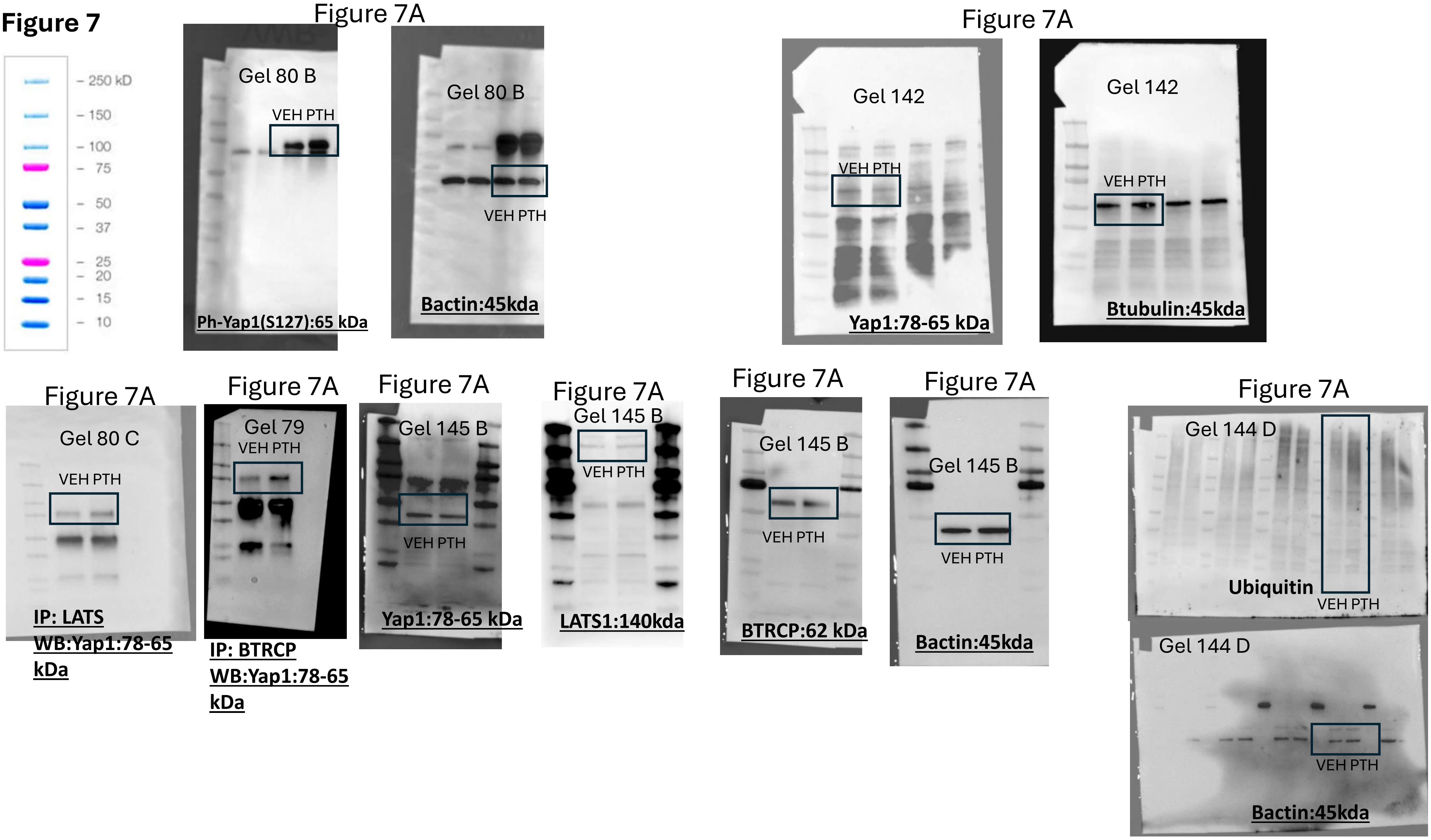

Figure 7A
250 kD
150
100
75
50
37
25
20
15
10
Gel 80 B
VEH PTH
Ph-Yap1(S127):65 kDa
Figure 7A
Gel 80 B
VEH PTH
Bactin:45kda
Figure 7A
Gel 142
VEH PTH
Yap1:78-65 kDa
Gel 142
VEH PTH
Btubulin:45kda
Figure 7A
Gel 80 C
VEH PTH
IP: LATS
WB:Yap1:78-65 kDa
Figure 7A
Gel 79
VEH PTH
IP: BTRCP
WB:Yap1:78-65 kDa
Figure 7A
Gel 145 B
VEH PTH
Yap1:78-65 kDa
Figure 7A
Gel 145 B
VEH PTH
LATS1:140kda
Figure 7A
Gel 145 B
VEH PTH
BTRCP:62 kDa
Figure 7A
Gel 145 B
VEH PTH
Bactin:45kda
Figure 7A
Gel 144 D
Ubiquitin
VEH PTH
Gel 144 D
VEH PTH
Bactin:45kda

Figure 7B

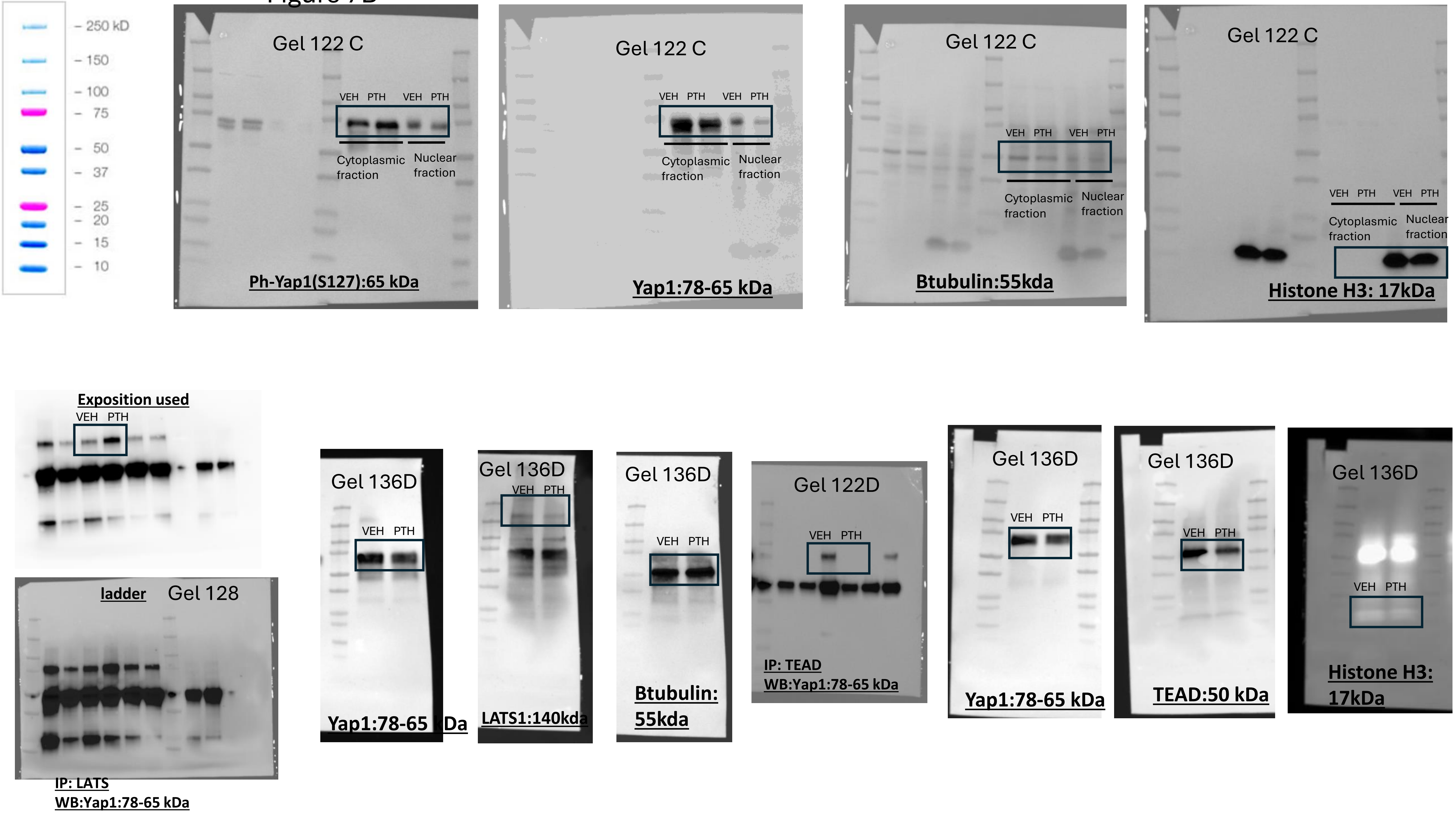

250 kD
150
100
75
50
37
25
20
15
10
Gel 122 C
VEH PTH VEH PTH
Cytoplasmic fraction
Nuclear fraction
Ph-Yap1(S127):65 kDa
Gel 122 C
VEH PTH VEH PTH
Cytoplasmic fraction
Nuclear fraction
Yap1:78-65 kDa
Gel 122 C
VEH PTH VEH PTH
Cytoplasmic fraction
Nuclear fraction
Btubulin:55kda
Gel 122 C
VEH PTH VEH PTH
Cytoplasmic fraction
Nuclear fraction
Histone H3: 17kDa
Exposition used
VEH PTH
ladder
Gel 128
IP: LATS
WB:Yap1:78-65 kDa
Gel 136D
VEH PTH
Yap1:78-65 kDa
Gel 136D
VEH PTH
LATS1:140kda
Gel 136D
VEH PTH
Btubulin: 55kda
Gel 122D
VEH PTH
IP: TEAD
WB:Yap1:78-65 kDa
Gel 136D
VEH PTH
Yap1:78-65 kDa
Gel 136D
VEH PTH
TEAD:50 kDa
Gel 136D
VEH PTH
Histone H3: 17kDa

Supplementary Figure 1

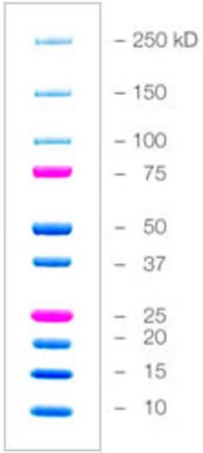

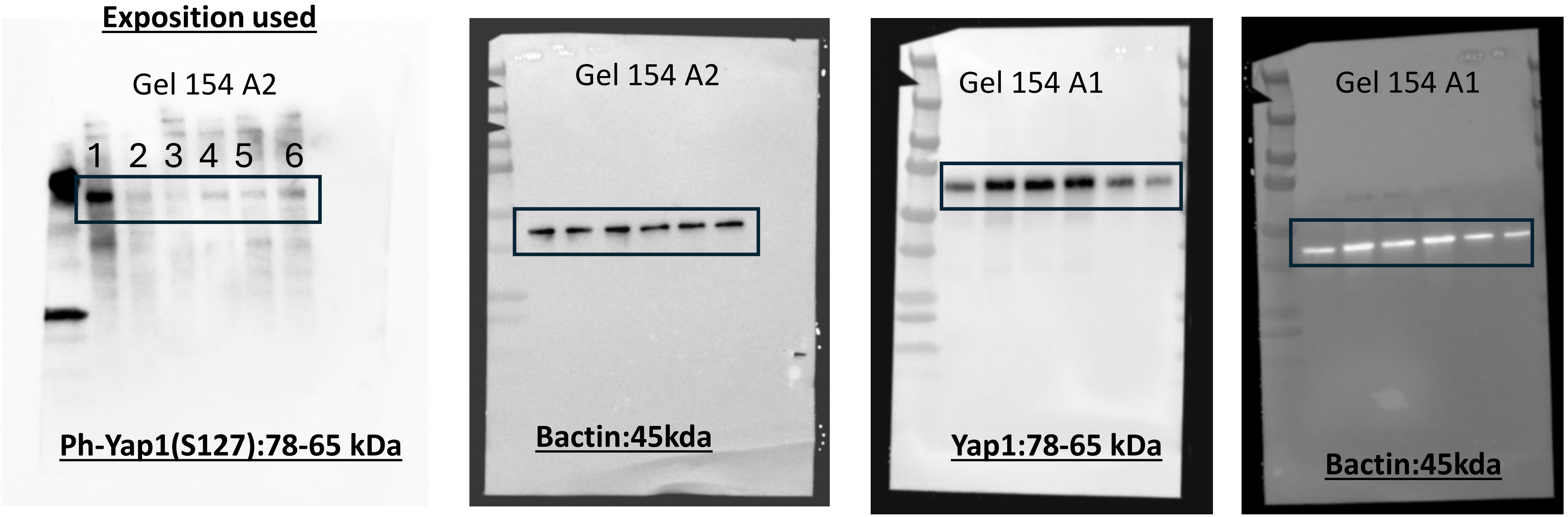

Loading:
Lane 1: CTR
Lane 2: PTH
Lane 3: PTH+H89
Lane 4: PTH+G0
Lane 5: H89
Lane 6:G0

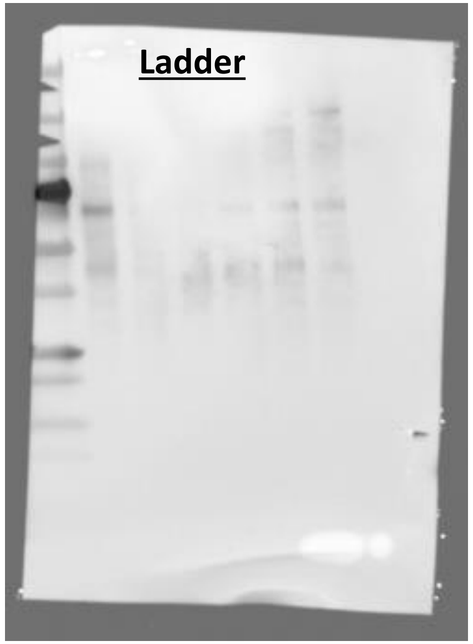

Supplementary Figure 2

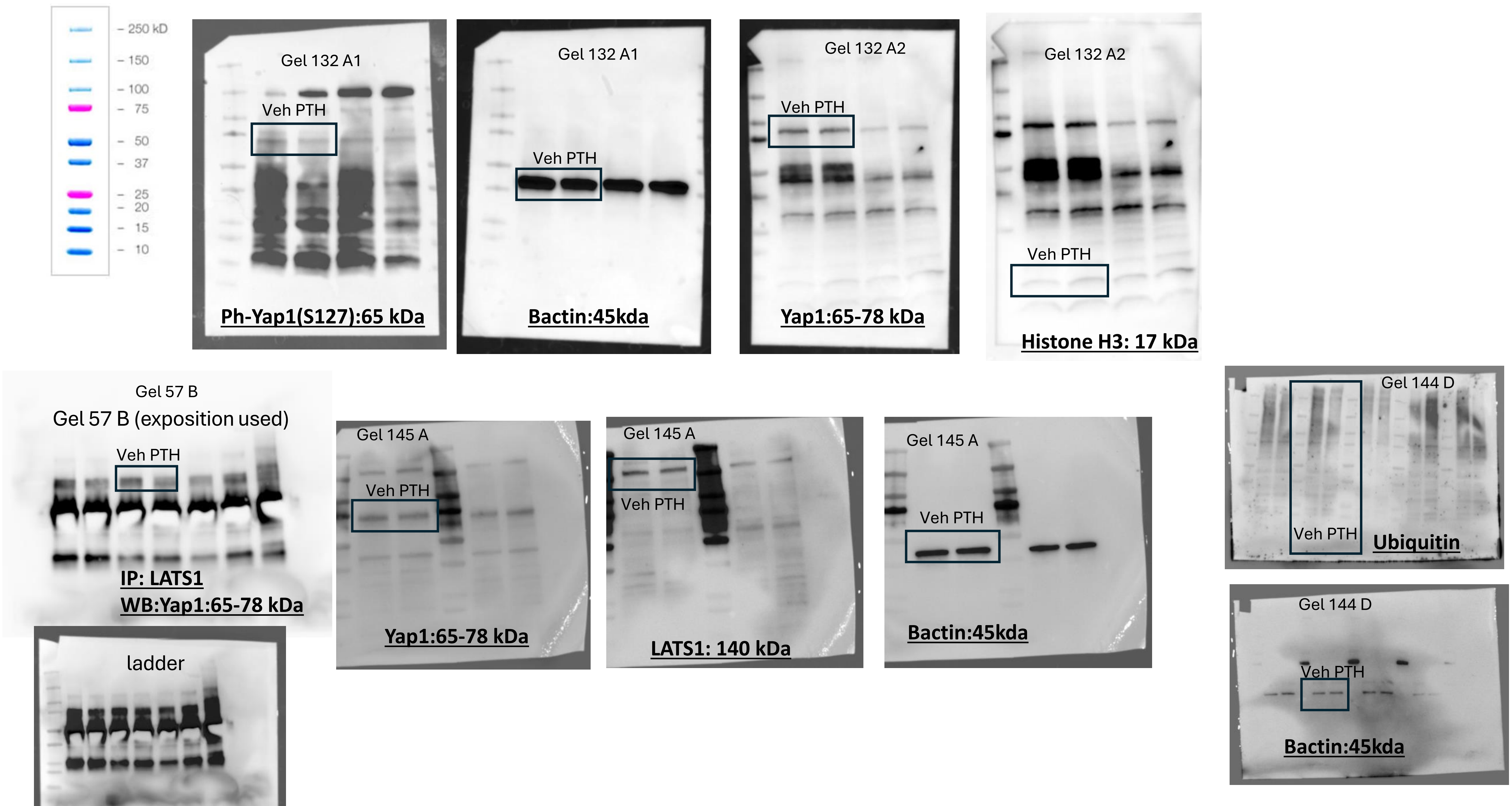

Supplementary Figure 3

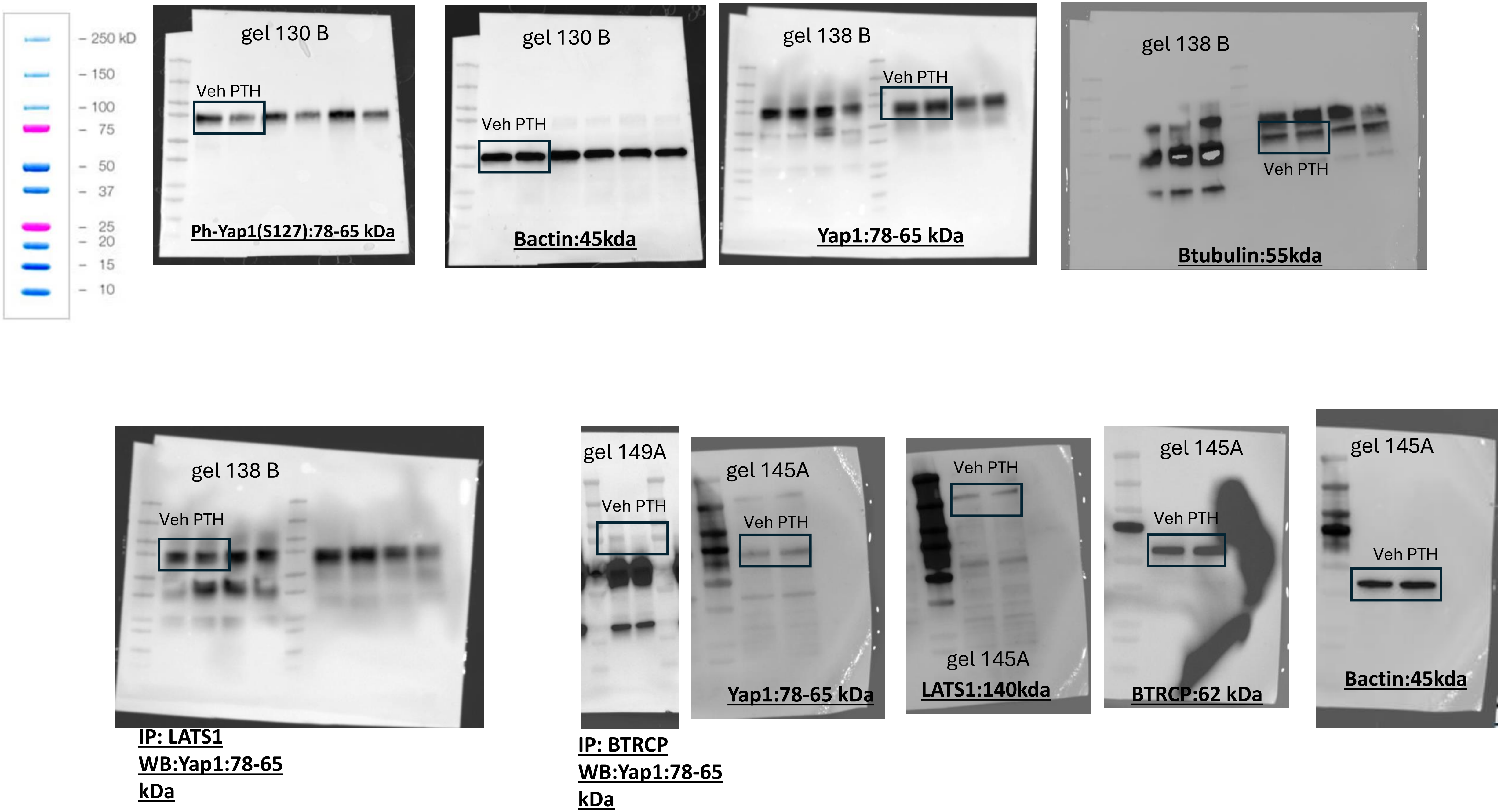

Supplementary Figure 3

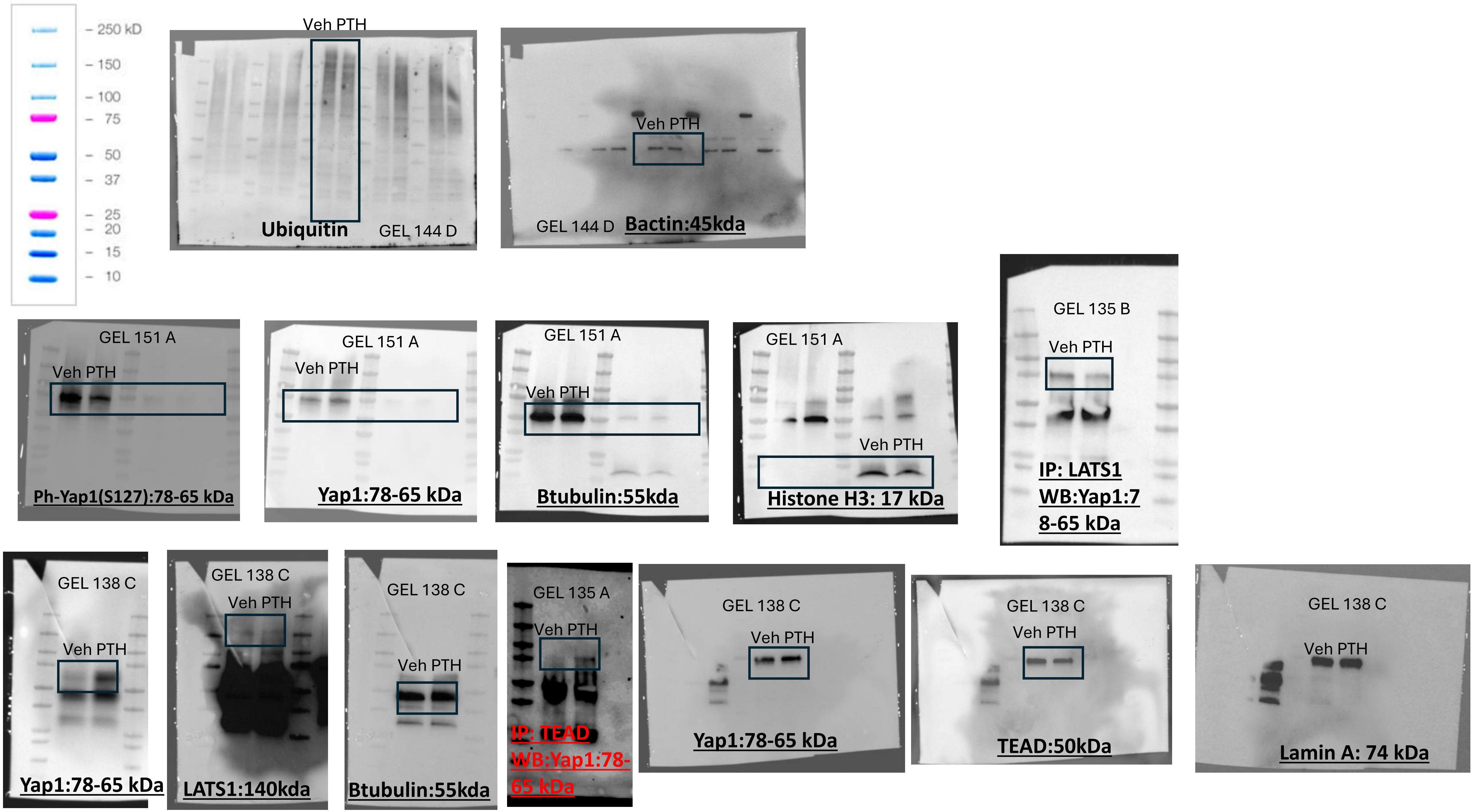

Supplement: Unedited blot and gel images [file jciinsight-10-191245-s171.pdf]
